# Development of a dual-functional conjugate of antigenic peptide and Fc-III mimetics (DCAF) for targeted antibody blocking†
†Electronic supplementary information (ESI) available. See DOI: 10.1039/c8sc05273e


**DOI:** 10.1039/c8sc05273e

**Published:** 2019-01-28

**Authors:** Lin Zhang, Hao Shen, Yiyi Gong, Xiaojing Pang, Meiqi Yi, Lin Guo, Jin Li, Sam Arroyo, Xin Lu, Sergey Ovchinnikov, Gong Cheng, Xudong Liu, Xu Jiang, Shan Feng, Haiteng Deng

**Affiliations:** a MOE Key Laboratory of Bioinformatics , Center for Synthetic and Systems Biology , School of Life Sciences , Tsinghua University , Beijing , China . Email: dht@mail.tsinghua.edu.cn; b Mass Spectrometry Facility , Westlake Lake University , Hangzhou , Zhejiang Province , China . Email: fengshan@westlake.edu.cn; c Institute for Protein Design , Department of Biochemistry , University of Washington , Seattle , WA , USA; d Central Research Laboratory , Peking Union Medical College Hospital , Chinese Academy of Medical Sciences and Peking Union Medical College , Beijing , China; e Tsinghua-Peking Center for Life Sciences , School of Medicine , Tsinghua University , Beijing , China; f Department of Biological Sciences , University of Notre Dame , South Bend , IN , USA

## Abstract

Targeted antibody blocking enables characterization of binding sites on immunoglobulin G (IgG), and can efficiently eliminate harmful antibodies from organisms. In this report, we present a novel peptide—denoted as a dual-functional conjugate of antigenic peptide and Fc-III mimetics (DCAF)—for targeted blocking of antibodies. Synthesis of DCAF was achieved by native chemical ligation, and the molecule consists of three functional parts: a specific antigenic peptide, a linker and the Fc-III mimetic peptide, which has a high affinity toward the Fc region of IgG molecules. We demonstrate that DCAF binds the cognate antibody with high selectivity by simultaneously binding to the Fab and Fc regions of IgG. Animal experiments revealed that DCAF molecules diminish the antibody-dependent enhancement effect in a dengue virus infection model, and rescue the acetylcholine receptor by inhibiting the complement cascade in a *myasthenia gravis* model. These results suggest that DCAFs could have utility in the development of new therapeutics against harmful antibodies.

## Abstract 2

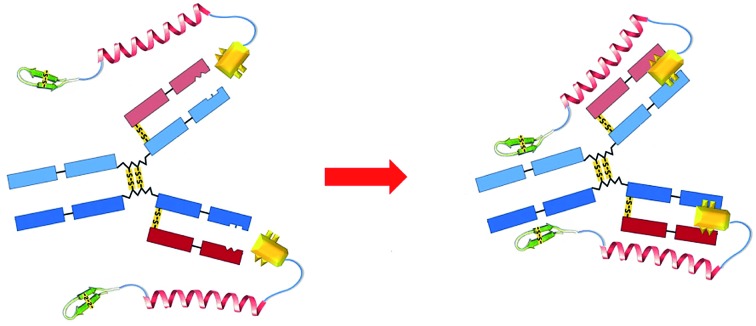
Long peptide DCAF enables high selectivity to target harmful antibodies, providing new thoughts for antibody-induced disease intervention.

## Introduction

Targeted therapies were first described by Paul Ehrlich in the 1900s and have since been studied widely and used to treat cancer. Targeted therapies enable enlargement of the therapeutic window by offering increased efficacy and decreased toxicity compared with regular drugs at the same dose.^1^

**Scheme 1:**
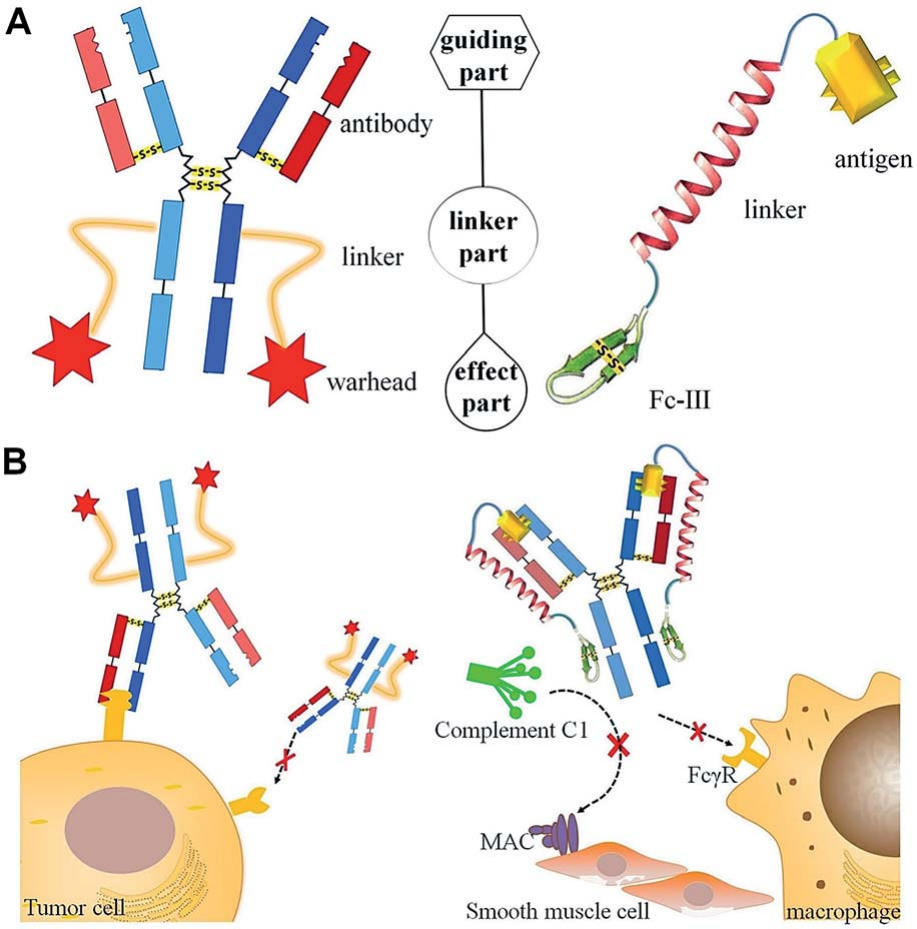
Comparisons of the structures (A) and functions for targeted therapy (B) of ADC (left) and DCAF (right). (A) The molecules for targeted therapy comprise a guiding part for specific recognition, a linker part and an effect part, which induces toxicity by inhibiting physiological processes. (B) Specific marker proteins on the cell membrane are recognized by ADC (left), while DCAF can target and block the cognate antibody on its Fc region to inhibit either Fc receptor (FcγR) binding or complement C1 component recruiting (right).

Over recent years, antibody-drug conjugates (ADCs) have developed rapidly. These are an innovative class of biopharmaceutical drugs, designed as targeted therapy for cancer.^2^ An ADC is composed of three elements: a highly selective antibody, a potent toxic drug (warhead) and a linker that conjugates the antibody with the warhead (Scheme 1A, left). Therefore, ADCs can selectively destroy cancer cells by combining the specificity of an antibody, which recognizes a target protein on the cancer cells, with the potency of a highly cytotoxic agent (Scheme 1B, left).^2^,^3^ The antibody of an ADC acts as a guide for specific antigen recognition; however, specific antigens may also target the cognate antibody. In serum and some tissues, there are thousands of antibodies with different epitopes. Some epitopes are harmful, such as over-reactive antibodies in auto-immune diseases (*e.g.*, *myasthenia gravis*)^4^ and cross-reactive antibodies in the antibody-dependent enhancement (ADE) process that occurs during dengue virus (DENV) infection.^5^ Therefore, elimination of harmful antibodies will aid the treatment of antibody-induced diseases.

Immunoglobulin G (IgG) contains multiple binding sites that are involved in a number of processes such as antigen recognition, nucleotide binding, complement cascade and Fc receptor interaction. The development of targeted antibody blocking not only expands our knowledge about the relevance of different binding sites on IgGs, but also allows the chemical diversity to be explored in order to develop new approaches for antibody-induced disease intervention. In this report, we utilized the design of an ADC molecule to develop a dual-functional conjugate of an antigenic peptide and Fc-III mimetics (DCAF) reagent to block antibodies. The DCAF molecule has three parts: an antigen peptide, an Fc-III or Fc-III-4C cyclic peptide and a long linker that conjugates the two peptides (Scheme 1A, right). The antigen peptide specifically recognizes the antibody to be inhibited. The Fc-III or Fc-III-4C cyclic peptide, which is composed of one or two intra-chain disulfide bonds,^6^ can be considered as the counterpart of the warhead in an ADC molecule and binds strongly to the Fc region of the antibody. The peptide binds with a different affinity, and thus competes with the Fc receptor on the cell surface or the complement C1 protein complex (Scheme 1B, right). This can prevent the ADE process during DENV infection and formation of the membrane attack complex (MAC) in *myasthenia gravis*. Because specific antibodies can bring highly toxic warheads to targeted cells to kill tumors while reducing toxicity toward other tissues, we expect that specific antigens will also cause the Fc-III mimetic peptide to block a cognate antibody. Therefore, the DCAF molecule should have minimum adverse effects on other antibodies.

## Results and discussion

### Design and structure simulation of DCAF molecules

**Fig. 1:**
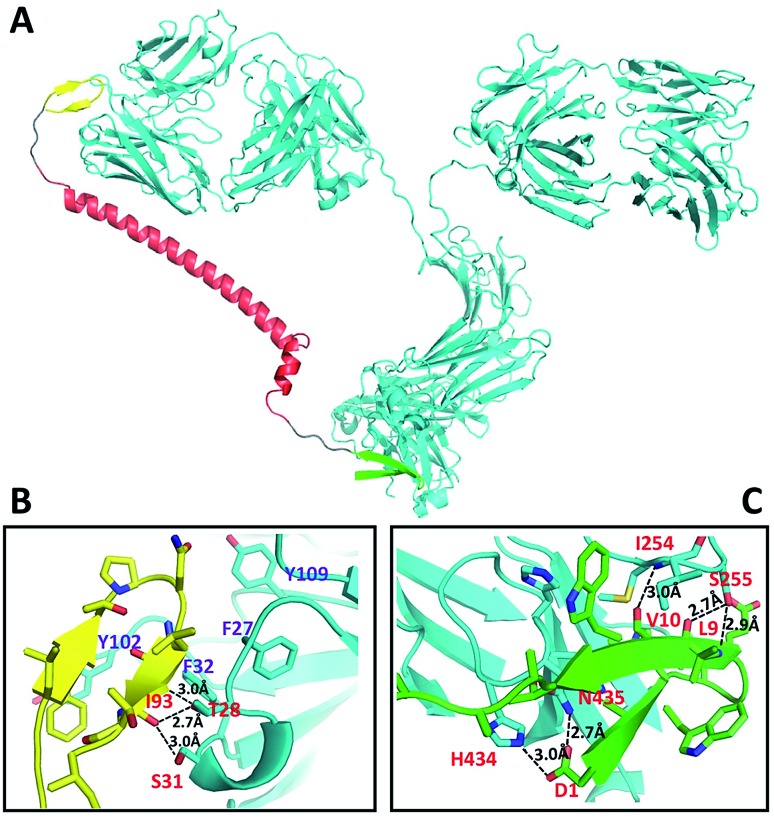
Structural representation of DCAF1 binding to IgG (A), and detailed analysis of the hydrogen bonds and ionic interactions of the antigen–antibody interaction including I93 to T28 and S31 (B) and the Fc-III-IgG complex including D1 to H434 and N435, L9 to S255 and V10 to I254 (C). The IgG molecule (cyan) is chiasmata-constructed from PDB IDs 4KVC and ; 1IGT; DCAF1 is composed of the antigen part (yellow), linker (red) and Fc-III fragment (green).

The DCAF molecule was created by conjugating the antigenic peptide with the Fc-III or Fc-III-4C tag using a 62 amino acid fragment of the Moesin FERM domain as the main part (α-helix) of the linker.^7^ Using a hydrophilic fragment of the α-helix from a solved structure as the linker had advantages over traditional chemical linkers such as increased bio-compatibility and orientated structure, and also enabled structure simulation that optimized the conformation of full-length DCAF. In brief, Rosetta^8^ was employed to optimize the structure of DCAF, which ensured that the antigen and Fc-III mimetics could bind to IgG simultaneously (see the Experimental). The GLFTPNLITI peptide, denoted as *pep*1, is a hybrid epitope from the E and prM proteins on the DENV surface. *Pep*1 interacts with 2H2 and 4G2 ADE-inducible mouse monoclonal antibodies.^9^*Pep*1 and the Fc-III fragments were chosen for assembly and structure simulation of DCAF1. The crystal structures of 2H2 Fab (PDB ID: ; 4KVC) and Fc-III bound to the Fc region complex (PDB ID: ; 1DN2), whose highly conserved structure with the whole IgG molecule (PDB ID: ; 1IGT) is demonstrated by superposition (Fig. S1†), were used to simplify the process of simulation and establish the model.^8^,^10^ The optimized structure of DCAF1 with two five-residue loops that conjugate the antigen, a long helix linker and Fc-III is shown in Fig. 1A; in which the antigen part (yellow) and Fc-III part (green) of DCAF1 bind the Fab and Fc regions of IgG, respectively. The total linker including the flexible loops was ∼11 nm long. Detailed hydrogen bond and salt bridge interactions of the antigen–antibody and Fc-III-antibody interfaces are displayed in Fig. 1B and C. The high binding affinity of the antigen–antibody pair is mediated mostly by hydrophobic interactions with the side chains of F27, F32, Y102 and Y109 of IgG; while the Fc-III-antibody complex is mainly restrained by hydrogen bonds (see Fig. 1 legend).

### Synthesis and characterization of DCAF

**Fig. 2:**
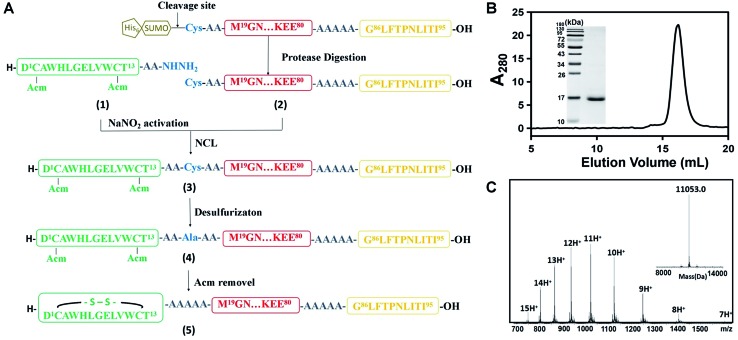
Chemical synthesis of the DCAF1 molecule. (A) The synthesis route by native chemical ligation. Different colors represent various parts of DCAF, consistent with Scheme 1. The antigen part is yellow, linker is red and Fc-III fragment is green. (B) Characterization of purified peptide **5** by SEC and SDS-PAGE (left, molecular weight ladder; right, peptide **5**). The calculated final purity of DCAF1 was 97.5%. (C) The products of peptide **5** were detected using a Synapt G2 Si (Waters) LC-MS, and the deconvoluted molecular weights is 11 053.0.

As described above, we synthesized four DCAFs with different antigenic peptides and Fc-III mimetics (Table S1†) to investigate the general chemical properties of DCAF binding to IgG. Due to the disulfide bond in the Fc-III or Fc-III-4C part, we used a semi-synthetic strategy to generate DCAFs; specifically, a modified native chemical ligation (NCL) approach for expressed protein ligation.^11^Fig. 2A shows the synthesis flowchart for DCAF1. The Fc-III peptide (**1**)—whose two thiol groups were protected by acetamidomethyl (Acm)—with hydrazine at its C-terminus, was synthesized through a solid phase peptide synthesis (SPPS). The product was purified and detected by MS with a monoisotopic peak at 915.92 Da for the doubly-charged molecule (Fig. S2A†). The linker and antigen parts (**2**) were fused with a SUMO tag at the N-terminus and recombinantly expressed and purified. Following the SUMO cleavage; peptide **2**, in which the Cys residue at the N-terminus was exposed, was further purified and identified (Fig. S2B†). Subsequently, peptides **1** and **2** were ligated by NCL (monitored by HPLC shown in Fig. S2C†) to generate peptide **3** (Fig. S2D†). Peptide **3** was then desulfurized (to form **4**, Fig. S2E†) and oxidized to form the disulfide bond (deprotection of Acm) in the Fc-III part. The final product of DCAF1 (**5**) was obtained through further refolding and purification and was characterized by SEC, SDS-PAGE and MS (Fig. 2B and C). Circular dichroism (CD) spectroscopy verified that there were no significant structural differences of the linker parts before and after NCL and refolding (Fig. S2F†).

Since the molecular weight of the final product (**5**) was only 2 Da lower than the reduced molecule, which contains two free thiol groups, bottom-up MS was used to verify disulfide bond formation. After trypsin digestion, the Fc-III-containing peptide in **5** was identified as the monoisotopic peak at 1006.45 *m*/*z* with a triple charge (Fig. S3A†). This peak shifted to 1045.13 *m*/*z* after reduction and alkylation, which was achieved using iodoacetamide and generated carbamidomethyl modified Cys residues (+57.02 Da) (Fig. S3B†), demonstrating that the Fc-III part of DCAF1 was disulfide bridged. The MS/MS spectra of non-reducing and alkylating peaks are shown in Fig. S3C and S3D,† respectively. Finally, all four DCAFs were synthesized using a similar approach to that shown in Fig. 2A. The molecular weights of the final products and all intermediates of DCAF2, DCAF3 and DCAF4 were detected by high-resolution MS (Fig. S4–S6†). The purities of all four products were estimated to be over 95% by size exclusion chromatography (SEC) and SDS-PAGE assays.

### Biophysical and biochemical assays of DCAF

The interactions between DCAF molecules and antibodies were evaluated by surface plasmon resonance (SPR) analysis. *Pep*1 showed high binding affinity toward the 4G2 antibody, denoted as *Ab*1,^9^ and the monoclonal antibodies *Ab*2 and *Ab*3 were generated by immunization of mice with *pep*2 and *pep*3 (see Table S1†), respectively. Both *pep*1 and the Fc-III peptide showed high binding affinities, with different *K*_d_ and kinetic values toward *Ab*1 immobilized on a CM5 chip (Fig. S7A and B†). When DCAF1 was added, the SPR curve appeared to be a mixed pattern of the *pep*1 and Fc-III peptide curves (Fig. S7C†). The apparent *K*_d_ value was calculated to be 55 nM, and bivalent dissociation values of *K*_d1_ and *K*_d2_ were 0.59 nM and 0.54 μM, respectively, indicating that the IgG molecule interacts with the antigen and Fc-III parts of DCAF. When DCAF2 (with *pep*2 as the antigen part) was reacted with *Ab*1, the SPR curve and *K*_d_ value (0.28 μM) were exactly the same as when the Fc-III peptide was included (Fig. S7D†). Similar observations were also made when *Ab*2, *Ab*3 and *Ab*4 were immobilized (all *K*_d_ values for thermodynamic parameters are listed in Table 1). Though the apparent dissociation constant of DCAF with its cognate *Ab* was larger than that of *pep* with *Ab*, the Gibbs free energy change of DCAF binding to its cognate *Ab* was still larger due to bivalent interactions. Fig. S7E† summarizes the kinetic information of DCAF 1–4 binding to their cognate IgGs. All of the antigen–antibody interactions exhibited fast binding rates (high *k*_a_) and slow dissociation rates (low *k*_d_), whereas the Fc-III parts and Fc binding regions exhibited high *k*_a_ and *k*_d_ values except for Fc-III-4C. Regarding stoichiometry, SEC experiments demonstrated that 1 M *Ab*1 could bind 2 M DCAF1, as the IgG peak shifted when the complex formed (Fig. S8†). From the SPR and SEC data, we concluded that one IgG molecule binds two DCAF molecules only—possibly due to steric hindrance—each of which can block both the antigen-binding part and Fc region of the antibody simultaneously, as outlined in Scheme 1.

**Table 1:** Summary of the *K*_d_ values (nM) of antibodies binding to Fc-III mimetics, their relevant antigens and DCAF molecules^*a*^

	*K*_d_ (nM)		*K*_d_ (nM)
*Ab*1 to *pep*1	0.47 ± 0.02	*Ab*3 to *pep*3	0.43 ± 0.02
*Ab*1 to Fc-III	239 ± 2	*Ab*3 to Fc-III	134 ± 6
*Ab*1 to DCAF1	55 ± 4	*Ab*3 to DCAF3	21 ± 1
*Ab*2 to *pep*2	1.02 ± 0.09	*Ab*4 to *pep*4	1.5 ± 0.3
*Ab*2 to Fc-III	169 ± 18	*Ab*4 to Fc-III-4C	13.99 ± 0.03
*Ab*2 to DCAF2	19.8 ± 0.8	*Ab*4 to DCAF4	6.56 ± 0.08

^*a*^All data represent mean ± SD (*n* = 3).

**Fig. 3:**
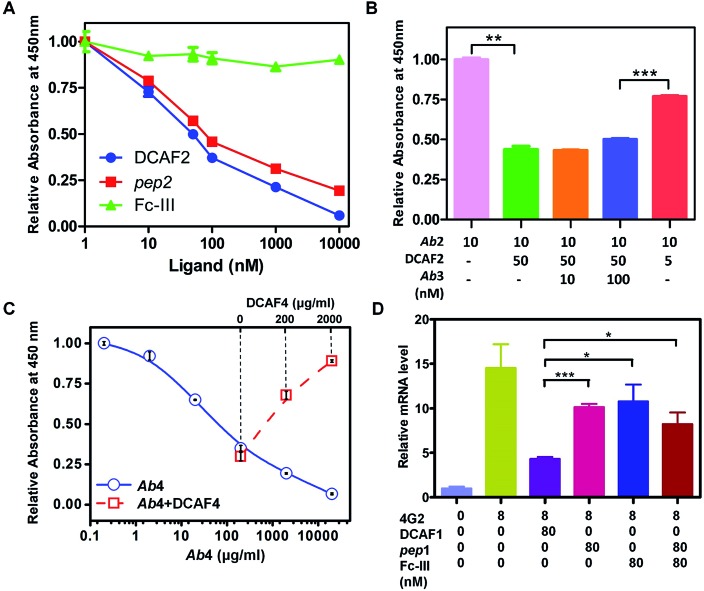
ELISA and ADE assays of DCAF molecules. (A) ELISA assay of *pep*2 (red squares), Fc-III (green triangles) and DCAF2 (blue circles) demonstrating inhibition of *Ab*2 binding using different ligand concentrations. (B) ELISA assay of irrelevant antibody *Ab*3 competing with DCAF2. (C) ELISA assays of *Ab*4 inhibiting C1q binding under different ligand concentrations and DCAF4 competition. (D) Relative mRNA levels of DENV2 in K562 cells measured by QRT-PCR. Data are shown as means ± SD from three experiments, * indicates *p* < 0.05, ** indicates *p* < 0.01 and *** indicates *p* < 0.001.

An ELISA competition assay was performed to evaluate the targeting effect of the DCAF molecule toward a cognate antibody. The sandwich method was used to immobilize 10 nM GST-fused *pep*2 on a well plate. *Ab*2 with different concentrations of inhibitors (DCAF2, Fc-III or *pep*2) was then added for colorimetric analysis. Both DCAF2 and *pep*2 significantly blocked *Ab*2 binding, with IC_50_ values at around 50 nM, consistent with the SPR results. The Fc-III tag did not inhibit antigen–antibody binding (Fig. 3A). When a higher dose of irrelevant antibody *Ab*3 was added to compete with the DCAF2 molecule, no significant changes were observed compared with *Ab*2 alone (Fig. 3B, columns 2–4). If DCAF2 was bound to all antibodies without selectivity, there would be no differences between the *Ab*3 and *Ab*2 groups when using the same ratio of the total antibody to DCAF2. Inhibition of *Ab*2 was less significant when 5 nM DCAF2 was used with 10 nM *Ab*2 than when 50 nM DCAF2 was used with 10 nM *Ab*2 or 100 nM *Ab*3 (Fig. 3B, columns 4–5). This demonstrates that DCAF2 binds more strongly to *Ab*2 than to other antibodies and thus minimizes off-target effects.

Binding between the Fc-III mimetic of DCAF and the Fc region of the antibody was examined to confirm whether this interaction blocks the binding of the Fc region with the Fc receptor or complement protein. Synthesis of DCAF4 was achieved by conjugating the Fc-III-4C tag and *pep*4—a 39-mer antigenic peptide^12^ that was selected to inhibit *Ab*4—the commercial antibody mAb35 that blocks the acetylcholine receptor (AChR) and induces *myasthenia gravis*. The ELISA assay for the C1q protein, which initiates the complement cascade, showed that *Ab*4 directly interacts with C1q, which is consistent with a previous study^13^ (Fig. 3C, blue solid line). The absorbance at 450 nm increased significantly when excess DCAF4 was added (Fig. 3C, red dashed line), indicating that DCAF4 can competitively inhibit the binding of C1q complement protein to *Ab*4. This provides an important support for DCAF4-based treatment of *myasthenia gravis.*

### Application 1: inhibiting the antibody-dependent enhancement effect in dengue fever

As described above, DCAF1 specifically targeted the 4G2 antibody (*Ab*1), which is an ADE effect-inducing antibody during DENV infection. As a subclass of the yellow fever virus, DENV is composed of four serotypes and is spread by several species of mosquito.^14^ Recently, a partially effective vaccine for dengue fever was developed using a combination of both yellow fever virus and four serotypes of DENV, which has become commercially available in some developing countries.^15^ Concerns for the vaccine include its ineffectiveness for all serotypes and the significant risk that the ADE process may bring serious symptoms such as hemorrhagic fever.^16^ The ADE effect is caused by cross-reactive antibodies generated by different serotypes of DENV and may enable virus entry into Fc receptor-presenting cells, increasing DENV infection.^5^,^17^ The elimination of cross-reactive antibodies that contribute to the ADE effect is a novel approach for controlling DENV infection.

To validate whether synthesized DCAF1 can decrease the ADE effect during DENV infection, *in vitro* ADE measurements were taken following administration of 4G2 to the K562 cell line, which had been infected with type 2 DENV. The RNA level of DENV2 increased ∼15-fold when 4G2 was added (Fig. 3D). When *pep*1 and Fc-III were co-incubated, a drop of approximately 30–35% in the reverse-transcript virus RNA level was observed. This suggested that either the antigen part or the Fc-III part inhibits the ADE process to some extent, blocking the antibody–virus or antibody–Fc receptor interaction, respectively. Consistent with previous results,^18^ reducing the affinity of the Fc region binding to the Fc receptor can therefore attenuate ADE. When the same concentration of DCAF1 was used, virus RNA levels decreased to less than 30% (Fig. 3D). An ADE competition assay was used to investigate excess irrelevant antigen (*pep*2) and DCAF2 incubation, revealing that they did not interfere with the DCAF1 treatment (Fig. S9A†). Because the DCAF molecule can inhibit antibody–virus binding and the antibody–Fc receptor interaction simultaneously, it is a more powerful agent to block ADE than either antigen peptides, the Fc-III peptide or a combination of the two (Fig. S9B†).

### Application 2: relieving symptoms of myasthenia gravis

The design of DCAF4 was intended to block the mAb35 antibody (*Ab*4), which blocks AChR^19^ and induces the passive transfer model of *myasthenia gravis* in Lewis rats.^20^*Myasthenia gravis* is an autoimmune disease caused by production of excessive antibodies, which attack AChR between the cell–cell junctions in nerve and muscle tissues.^21^ In brief, AChR autoantibodies impair nerve conduction to prevent muscle contractions mainly through three mechanisms: (i) directly blocking acetylcholine binding to AChR; (ii) accelerating internalization and degradation of AChR; and (iii) recruiting complement components to form the membrane attack complex (MAC) and destroy the neuromuscular junction (NMJ). The effects of the autoantibodies are largely dependent on activation of the complement cascade.^22^ Although different intervention strategies such as cholinesterase inhibitors, corticosteroids, immunosuppressive therapy and thymectomy have been developed for *myasthenia gravis* treatment,^23^ an effective therapy with few adverse effects is urgently needed. In the present work, we delivered DCAFs to block AChR autoantibodies.

**Fig. 4:**
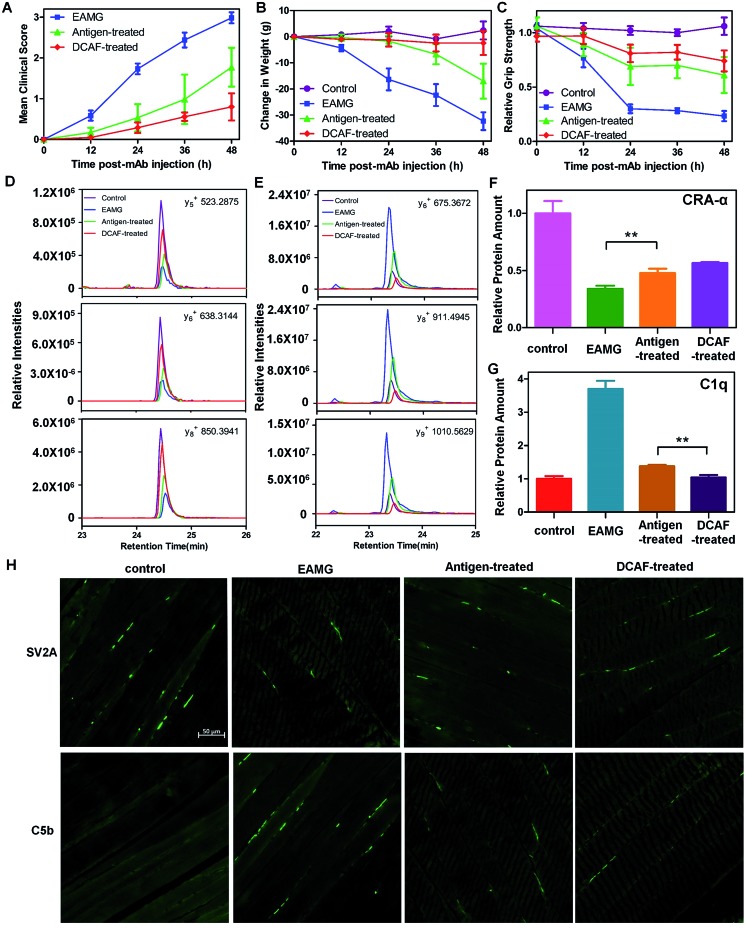
Application of DCAF4 in the rat EAMG model. (A–C) Clinical scores, weight loss and relative grip strength in the control (purple), EAMG (blue), antigen-treated (green) and DCAF-treated groups (red) at 0, 12, 24, 36 and 48 h after injection. For each group, *n* = 5. (D) The intensities of y_5_, y_6_ and y_8_ ions of WNPDDYGGVK from CRA-α in the control (purple), EAMG (blue), antigen-treated (green) and DCAF-treated groups (red). (E) The intensities of y_6_, y_8_ and y_9_ ions of LEQEEVVHLQATDK from C1q in the control (purple), EAMG (blue), antigen-treated (green) and DCAF-treated groups (red). (F–G) Normalized abundance of CRA-α and C1q in the four groups using two reference peptides from GAPDH. (H) Representative SV2A (top) and C5b (bottom) immuno-histofluorescence images of the rat tibialis anterior muscle from the control, EAMG, antigen-treated and DCAF-treated groups. Data are shown as means ± SD from the three experiments, where ** indicates *p* < 0.01.

We established an experimental autoimmune *myasthenia gravis* (EAMG) model in the Lewis rat by intraperitoneal injection of *Ab*4, as described in a previous study.^24^ To determine whether DCAF4 blocked the activity of mAb35 in the rat model and caused remission of the symptoms, Lewis rats were treated with either PBS (control group), the mAb35 antibody (EAMG group), mAb35 followed by *pep*4 (antigen-treated group) and mAb35 followed by DCAF4 (DCAF-treated group). Rats with more serious symptoms were given a higher clinical score and had lower weights and weaker grip strength. During the 48 h treatment, we observed that both the antigen-treated and DCAF-treated groups exhibited reduced clinical symptoms and weight loss, and gained grip strength compared with the EAMG group (Fig. 4A–C). Rats from the DCAF-treated group showed the most significant recovery.

Furthermore, we evaluated the effects of *pep*4 and DCAF4 on the levels of the cholinergic receptor, nicotinic, α1 (CRA-α, a kind of AChR) and C1q proteins in the tibialis anterior muscle using the parallel reaction monitoring (PRM) method. If *pep*4 and DCAF4 inhibited *Ab*4 to relieve the symptoms, higher levels of AchR could be expected in muscle tissue. Subsequently, changes in the levels of C1q could indicate whether any increase in AchR was due to complement system pathway blockade. For each protein, three peptides were selected as representative peptides for protein quantification (see the Experimental), and three fragmented ions of each peptide were used for quantitation. For example, the intensities of y_5_, y_6_ and y_8_ were monitored for WNPDDYGGVK in CRA-α (Fig. 4D); and the intensities of y_6_, y_8_ and y_9_ were monitored for LEQEEVVHLQATDK in C1q (Fig. 4E). The results showed that *pep*4 and DCAF4 caused CRA-α levels to increase by 41% and 67%, respectively, compared with those of the EAMG group (Fig. 4F). Decreases of 37% and 28% in the C1q levels were observed in the antigen-treated and DCAF-treated groups, respectively, compared with the EAMG group. Notably, the C1q level was 25% lower in the DCAF4 treated group than in the antigen-treated group (Fig. 4G), indicating that DCAF caused significant inhibition of the complement cascade, and restored AChR in muscle tissue. Immuno-histofluorescence was carried out to directly study the NMJ and MAC in rat muscle tissue, by preparing paraformaldehyde-embedded sections of the tibialis anterior muscle and staining with SV2A and C5b antibodies. The marker protein SV2A was used to identify synaptic vesicles where Ach signal transmission was occurring, while C5b was a complement component protein in the MAC. The intensity of fluorescence reflected the amount of protein in the visual field. As expected, less NMJ and more MAC signals were detected in the EAMG group than in the control group, whereas the DCAF4 treatment restored the NMJ level and reduced the MAC level to some extent (Fig. 4H). Comparing the DCAF-treated group with the antigen-treated group revealed a more significant change in the MAC or complement system than in the NMJ or AchR (Fig. S10†), in line with the PRM results. The less significant observations in these two groups might be induced by the variability of individuals, and differences in the molar amounts of the two reagents used.

## Conclusions

We designed and developed DCAF molecules that effectively targeted and inhibited the ADE process of DENV infection, and blocked autoantibodies against AChR in the neuromuscular disease *myasthenia gravis*. DCAFs simultaneously bound to the Fab and Fc regions of IgG, with a maximum stoichiometry of DCAF binding to IgG estimated to be 2 : 1. The guiding part (antigen) of DCAF leads to a targeted binding effect, and the Fc-III or Fc-III-4C part strongly prevents other Fc-binding molecules (such as complement protein C1q) from interacting with IgG. Translational studies revealed that DCAF1 blocked the 4G2 antibody with high efficacy and inhibited the ADE process during DENV infection in a cell line model, whereas DCAF4 relieved the disease symptoms by complement cascade inhibition and MAC reduction in a passive transfer *myasthenia gravis* rat model.

## Experimental

### Materials

Fmoc-amino acids and Wang resin were purchased from C S Bio, GL Biochem (Shanghai, China). 2-Chlorotrityl resin was purchased from Hecheng Technology (Tianjin, China). *N*,*N*-Diisopropyl-carbodiimide (DIC), ethyl cyanoglyoxylate-2-oxime (oxyma), triisopropylsilane (TIPS), trifluoroacetic acid (TFA) (HPLC grade), hydrazine hydrate 85%, silver acetate, *N*,*N′*-dimethylformamide (DMF) and CH_2_Cl_2_ (DCM) were purchased from J&K Scientific (Beijing, China). 4-Mercaptophenylacetic acid (MPAA) was purchased from Alfa Aesar. 2-Methyl-2-propanethiol (^*t*^BuSH) was purchased from Aladdin (Shanghai, China). 2,2′-Azobis[2-(2-imidazolin-2-yl)propane]dihydrochloride (VA-044) was purchased from yuanye Bio-Technology (Shanghai, China). Other related reagents were purchased from Beijing Chemical Works (Beijing, China) or Sinopharm Chemical Reagent. All solvents were of HPLC grade.

### Rat strain and cell culture

Female Lewis rats were obtained from the animal facility of Peking Union Medical College Hospital. All studies were approved under Institutional Animal Care and Use Committee (IACUC) of Peking Union Medical College Hospital. Cell line K562 was purchased from ATCC and grown in IMDM medium supplemented with 10% FBS and 1% penicillin/streptomycin at 37 °C in a humidified incubator with 5% CO_2_.

### Structure simulation

To simplify the process of simulation, the crystal structures of 2H2 Fab (4KVC) and Fc-III binding with a Fc region complex (; 1DN2) were used to establish the model.6a,^10^ Since the antigen doesn't have a solved structure, *ab initio* structure prediction was performed and the best 100 PatchDock models were refined using Rosetta. The lowest energy solution was strongly converged and yielded the antigen–antibody docking model.^25^ Then the antigen–antibody docking model, the structure of the Fc binding complex (; 1DN2), the connecting helix extracted from 1E5W, and the extracted antibody structure of IgG (; 1IGT) were connected and further refined in Rosetta.^8^

### Protein expression and purification

The linker and antigen parts (**2**) were constructed into the pET-28a plasmid fused with a His-tag and SUMO-tag at its N terminal, and expressed in *E. coli* BL21 (DE3)-plysS strain from Stratagene (Heidelberg, Germany) and purified. In brief, bacteria transformed with the plasmid were grown at 37 °C, when *A*_600_ reached the value 0.6, the protein expression was triggered by adding 0.5 mM IPTG overnight at 25 °C. After cell harvests, a lysis buffer (50 mM NaH_2_PO_4_, 300 mM NaCl, 10 mM imidazole, pH 8.0) was used to lyse cells. Cell lysates were sonicated on ice and centrifuged at 15000*g* for 30 min at 4 °C. The clear supernatants were purified by Ni-NTA agarose (Qiagen) followed by desalting using a HiTrap Desalting column (GE Healthcare). Then the protein was cleaved using SUMO protease with 1 mM DTT in cleavage buffer and further purified using a HiTrap SP column (GE Healthcare) to remove His-tag and SUMO-tag and Vydac C18 TP (Grace) to eliminate other contaminated proteins. The purities of the recombinant proteins were estimated to be over 95% by SDS-PAGE.

### Circular dichroism (CD) spectroscopy

CD spectra were recorded on a Pistar π-180 spectrometer from 260 nm to 195 nm at 25 °C in a quartz cell with 1 mm path length. The final concentration of the samples was about 10 μM. The spectrum for each peptide was obtained in triplicate, averaged, subtracted from blank and smoothed.

### Synthesis of peptides with a Solid Phase Peptide Synthesis (SPPS)

All the peptides were synthesized using standard Fmoc SPPS protocols under microwave conditions (CEM Liberty Blue) starting from Wang resin or 2-Cl-(Trt)-NHNH_2_ resin. The side-chain AAs were Arg(Pbf), Asn(Trt), Asp(O*t*Bu), Glu(O*t*Bu), His(Trt), Lys(Boc), Ser(*t*Bu), Thr(*t*Bu), Trp(Boc), Tyr(*t*Bu), and Cys(Acm). Firstly, 2-Cl-(Trt)-Cl resin was treated with 5% NH_2_NH_2_ in DMF (30 min for twice) to prepare 2-Cl-(Trt)-NHNH_2_ resin.11*c* This resin was next used to prepare the corresponding peptide hydrazides. The peptide chain elongation was carried out by using standard deprotection and coupling cycles under microwave conditions. After the completion of SPPS, the peptide was cleaved from the resin with cocktails TFA/DODT/TIPS/H_2_O (92.5/2.5/2.5/2.5) at room temperature for about 2 hours, and isolated by RP-HPLC to obtain the target peptide.

### Native chemical ligation

The C-terminal peptide hydrazide was dissolved in acidified ligation buffer (aqueous solution of 6 M GnHCl and 0.2 M NaH_2_PO_4_, pH 3.0). The mixture was cooled in an ice-salt bath (–15 °C) for 15 min, and 10 eq. NaNO_2_ in acidified ligation buffer was added. The activation reaction system was kept in an ice-salt bath with stirring for 15 min, after which 40 eq. MPAA in acidified ligation solution with 1.2 eq. N-terminal Cys peptide was added. The mixture was kept in an ice-salt bath for 5 min under stirring, after which the pH of the solution was adjusted to 6.8–7.0 at room temperature. After 8 h, 30 mM tris(2-carboxyethyl)phosphine hydrochloride (TCEP) in ligation buffer (pH 7.0) was added to dilute the system and the reaction system was kept for 20 min under stirring. Finally, the ligation product was analyzed and purified by RP HPLC and MS. The final products of DCAF 1 and 3 were further purified by SEC using a Superdex 200 Increase column, and the purities of all the DCAF molecules were estimated to be over 95% by SEC assay and SDS-PAGE.

### Desulfurization

The ligation product was dissolved in phosphate neutral buffer (pH 7.0) containing 6.0 M GnHCl, and 0.2 M NaH_2_PO_4_. To the above solution, 0.5 M TCEP, 10% ^*t*^BuSH and 10 eq. VA-044 solution (0.1 M in phosphate neutral buffer) were added. The final pH of the solution was adjusted to 6.9 and kept at 37 °C with stirring for about 8 hours. Then the product was purified by RP-HPLC and analyzed by MS.

### Removal of ACM

The resultant material was dissolved in H_2_O/CH_3_COOH (1 : 1). Then 40 eq. AgOAc was added to the solution, and the reaction was stirred at room temperature for 4 hours. Subsequently, 1 M dithiothreitol (DTT) dissolved in 0.2 M phosphate solution containing 6 M GnHCl (pH 7.0) was added to convert silver thiolate on proteins to free thiols; a precipitate was formed immediately. After being stirred violently, centrifugation was conducted, and the supernatant was purified and analyzed by RP-HPLC and MS.

### Sample preparation for proteomic analysis

For disulfide bond characterization, reducing or non-reducing SDS-PAGE was used in DCAF molecules. The sample was digested by trypsin with or without prior reduction (25 mM dithiotreitol, 55 °C, 45 min) but alkylation (55 mM iodoacetamide, 30 min in the dark) in 50 mM ammonium bicarbonate at 37 °C overnight. The digested products were extracted twice with 1% formic acid in 50% acetonitrile aqueous solution, and dried to reduce volume using Speedvac; For PRM analysis, the tibialis anterior muscles were homogenized and lysed in RIPA buffer and the protein concentrations were determined by the BCA method. 100 μg proteins for each group were used for in-solution trypsin digestion. Briefly, the samples were reduced with 10 mM dithiotreitol for 45 min at 50 °C and alkylated with 50 mM iodoacetamide for 20 min in the dark. Then immediately 4 volumes of pre-chilled (–20 °C) 100% acetone were added to the sample and incubated at –20 °C for one hour to precipitate proteins. Proteins were digested with sequencing grade modified trypsin for 16 h at 37 °C in 100 mM triethylammonium bicarbonate (TEAB). The digestion was stopped by thawing and refreezing, and then the sample was dried using a Speedvac and resuspended in 0.1% TFA.

### HPLC and mass spectrometry (MS)

Reverse phase HPLC was performed on a Thermo DIONEX ultimate 3000 ultra-high performance liquid chromatography system or Waters 2535 preparative liquid chromatography. For peptide analysis and purification, Vydac C18 TP (300 Å, 4.6 × 250 mm) and Vydac C18 TP (300 Å, 10 × 250 mm) columns were used at a flow rate of 1.0 mL min^–1^ or 4.72 mL min^–1^, respectively. The UV absorption at 214 nm was monitored for the injections. Mobile phase A consisted of 0.1% trichloroacetic acid in water, and mobile phase B consisted of acetonitrile containing 0.1% trichloroacetic acid. The high-resolution mass spectra were obtained on either a LTQ-Orbitrap Velos LC-MS (Thermo) or Synapt G2 Si LC-MS (Waters) for protein characterization.

### LC-MS/MS analysis

For LC-MS/MS analysis, the products were separated by 60 min gradient elution at a flow rate of 0.250 μL min^–1^ with a UltiMate™ 3000 RSLCnano system (Thermo Scientific) which was directly interfaced with a Thermo Q Exactive. The analytical column was a home-made fused silica capillary column (75 μm ID, 150 mm length; Upchurch, Oak Harbor, WA) packed with C-18 resin (300 Å, 5 μm, Varian, Lexington, MA). Mobile phase A consisted of 0.1% formic acid, and mobile phase B consisted of 100% acetonitrile and 0.1% formic acid. The Q Exactive mass spectrometer was operated in the data-dependent acquisition mode using Xcalibur 2.2.7 software and there was a single full-scan mass spectrum in the Orbitrap (400–1800 *m*/*z*, 30 000 resolution) followed by 20 data-dependent MS/MS scans in the ion trap at 35% normalized collision energy. Each mass spectrum was analyzed using a Thermo Xcalibur Qual Browser.

### Surface plasmon resonance measurement and affinity analysis

Surface plasmon resonance (SPR) experiments were performed using a BIAcore T200 instrument (GE healthcare) equipped with a CM5 sensor chip. Briefly, *Ab*1, *Ab*2 or *Ab*3 were directly immobilized onto a CM5 sensor chip using an EDC/NHS amine coupling kit. A blank channel was used as the negative control. For SPR measurements, all peptides were directly dissolved in running buffer (PBS with 0.005% surfactant P20). The analytes were injected and disassociated with immobilized IgG at a flow rate of 30 μl min^–1^. After each binding and dissociation cycle, the surface of a sensor chip was regenerated using 10 mM glycine-HCl buffer (pH 2.0). The sensorgrams were corrected by subtraction of the signal from the negative control flow path and curve-fitted to calculate the reaction rate and constants using Evaluation 1.0 software.

### Size exclusion chromatography analysis

The stoichiometry of DCAF to IgG is determined on an ÄKTA purifier 10 (GE Healthcare). A Superdex 200 Increase column was used to characterize the complex at a flow rate of 0.5 mL min^–1^ and the UV absorptions at 215 nm and 280 nm were monitored for the injections.

### Antibody dependent enhancement (ADE) assay

When taking the ADE blocking assay, a mixture of virus (0.1 MOI) and 4G2 (0.12 μg, 8 nM) was added to a 24-well plate containing K562 cells at 50 000 cells per well and incubated for 1 hour at 37 °C followed by addition of DCAF (0.1 μg, 80 nM) or the other inhibitors (80 nM). After culture at 37 °C for 3 days, total RNA was extracted from the infected cells using an AxyPrep Multisource Total RNA Miniprep Kit (Axygen, USA) and was subjected to one-step real-time PCR. Same procedures were used for a competitional ADE assay, *pep*2 (80 nM) or DCAF2 (80 nM), as the irrelevant antigen or DCAF molecule, *Ab*2 (8 nM or 80 nM), as the irrelevant antibody, were incubated with 4G2 (0.12 μg, 8 nM). The data derived from real-time PCR were analyzed by the ΔΔ*C*_t_ analysis method using GAPDH as an internal control. The assays were performed in triplicate and the results were expressed as averages with standard error.

### ELISA assay

A standard sandwich ELISA assay was used for competition experiment. Briefly, 96-well microtiter plates were coated overnight with anti-GST antibody (10 nM) at 4 °C and blocked with 200 μL of 1% BSA in PBS at room temperature for 1 h. Then the artificial antigen (0.5 nM), *pep*2 coupled with the GST protein, was added to the wells and incubated for 2 h at RT. The plates were washed with PBS/0.05% Tween 20, and *Ab*2 (10 nM) or *Ab*2 (10 nM) plus other ligands, antigen, Fc-III or DCAF2 at series concentrations, were added and incubated for 2 h at RT. After extensive washing, they were incubated for 0.5 h at room temperature with anti-rabbit IgG, HRP-linked antibody (Cell Signaling Technology, 7074, 1 : 20 000), and revealed by the TMB substrate and measured at OD_450_. For a C1q binding assay, rat C1q ELISA kit was used following the protocol of the manufacturer. Briefly, incubated 25 ng mL^–1^ C1q and series concentrations of *Ab*4 or DCAF4 with a pre-coated C1q capture antibody for 2 h at RT. Then HRP linked detection antibody was added for 1 h at RT and the TMB substrate was used to measure the concentration of C1q at OD_450_.

### Passive induction of experimental autoimmune myasthenia gravis (EAMG) and treatment with DCAF4

Female Lewis rats, 6–8 weeks of age, weighing approximately 180 g, were injected with mAb35 (BioXCell, BE0123) at 100 μg/100 g intraperitoneally (i.p.) in PBS to induce the experimental autoimmune *myasthenia gravis* (EAMG) model. Rats were assessed for onset of EAMG every 12 h, such as score for weakness, measured the weight loss and strength loss. Rats were divided into four matched groups, each comprising of five female Lewis rats. Each group was injected with mAb35 in PBS (i.p.), while the control group was injected with an equal amount of PBS (i.p.) instead of mAb35 and the drug treated group was injected with a mixture of mAb35 and *pep*4 (2.5 mg/100 g) or DCAF4 (2.5 mg/100 g). Clinical scores for animals are as follows: 0, no weakness and no abnormalities; (1) fatigable or weakness is only observed after exercise; (2) clinical signs of weakness present before exercise, loss of grip in front paws, hunched posture, or head down; (3) severe clinical signs of weakness: no ability to grip, hindlimb paralysis, weight loss>15%, immobility or moribund; (4) death.^24^

### Parallel Reaction Monitoring (PRM) assay

The tibialis anterior muscles were homogenized and lysed in RIPA buffer followed by reduction, alkylation and trypsin digestion. The digested peptides were injected into a Thermo Orbitrap Fusion Lumos using PRM mode with 40 min gradient. The isolation list contained two internal control peptides from GAPDH (GAAQNIIPASTGAAK and LISWYDNEYGYSNR), three peptides from the C1q complex (LEQEEVVHLQATDK, GLFQVLAGGTVLQLQR and FNSAITNPQGDYNTNTGK) and three peptides from CRA-α (WNPDDYGGVK, QQWVDYNLK and IHIPSEK). All the quantification experiments were performed in three replicates. The raw data were further analyzed by Skyline and the relative amounts for each protein were normalized by internal control.

### Immuno-histofluorecence staining

The rats were euthanized and the tibialis anterior muscles were obtained. Then the tissues were fixed in 4% paraformaldehyde and embedded in paraffin. The fixed tissue (5 μm) on the slides were de-paraffinized in xylene and rehydrated by graded alcohols, followed by antigen retrieval, which used citrate unmasking buffer at 95 °C, 30 min, then 115 °C, 30 s. After cooling down to RT, endogenous peroxidase activity was quenched by 3% H_2_O_2_ for 10 min. Subsequently, the slides were rinsed three times with distilled water, and transferred to PBS. The slides were then blocked with 5% goat serum in PBS for 1 h and incubated with primary antibodies for 1 h. Following extensive washing in PBS, corresponding fluorescent secondary antibodies were added for 1 h. Primary antibodies include mouse anti-SV2A (Santa Cruz Biotechnology, sc-376234, 1:100) and rabbit anti-C5b polyclonal antibody (ABclonal, A8104, 1:100), while secondary antibodies contain anti-mouse IgG (H + L) F(ab′)_2_ fragment (Alexa Fluor® 488 Conjugate) (Cell Signaling Technology, 4408, 1:500) and anti-rabbit IgG (H + L) F(ab′)_2_ fragment (Alexa Fluor® 488 Conjugate) (Cell Signaling Technology, 4412, 1:500).

## Author contributions

L. Z, S. F. and H. D. initiated the project. M. Y., H. S. and S. O. performed structure simulation. L. Z., S. F., L. G. and J. L. performed molecules synthesis and biochemical experiments. L. Z, S. F., X. P. and G. C. performed ADE inhibiting experiments. L. Z, S. F., Y. G., X. Lu, X. Liu and X. J performed EAMG experiments. S. A., S. F. and H. D. wrote the paper, and all authors approved the manuscript.

## Conflicts of interest

The authors declare no conflict of interests.

## Supplementary Material

Supplementary information
