# Supplementary material for: Development of a dual-functional conjugate of antigenic peptide and Fc-III mimetics (DCAF) for targeted antibody blocking
Source: Chem Sci. 2019 Jan 28;10(11):3271–80. doi: 10.1039/c8sc05273e (PMC6429600; doi:10.1039/c8sc05273e)
Supplement: Supplementary file 1 [file SC-010-C8SC05273E-s001.pdf]

Electronic Supplementary Material (ESI) for Chemical Science.
This journal is © The Royal Society of Chemistry 2019

Supporting Information

Development of a Dual-functional Conjugate of Antigenic Peptide and Fc-III Mimetics (DCAF) for Targeted Antibody Blocking

Lin Zhang,[a] Hao Shen,[c] Yiyi Gong,[d] Xiaojing Pang,[e] Meiqi Yi,[a] Lin Guo,[a] Jin Li,[a] Sam Arroyo,[f] Xin Lu,[f] Sergey Ovchinnikov,[c] Gong Cheng,[e] Xudong Liu,[d] Xu Jiang,[d] Shan Feng,*[b] Haiteng Deng*[a]

[a]MOE Key Laboratory of Bioinformatics, Center for Synthetic and Systems Biology, School of Life Sciences, Tsinghua University, Beijing, China.

[b]Mass Spectrometry Facility, Westlake Institute for Advanced Study, Hangzhou, Zhejiang Province, China.

[c]Institute for Protein Design, Department of Biochemistry, University of Washington, Seattle, WA., USA.

[d]Central Research Laboratory, Peking Union Medical College Hospital, Chinese Academy of Medical Sciences and Peking Union Medical College, Beijing, China.

[e]Tsinghua-Peking Center for Life Sciences, School of Medicine, Tsinghua University, Beijing, China.

[f]Department of Biological Sciences, University of Notre Dame, South Bend, IN., USA.

*e-mail: dht@mail.tsinghua.edu.cn

Chemical synthesis procedure

1. Synthesis of Fc-III mimetics:

The Fc-III peptide (**1**), DC(Acm)AWHLGELVWC(Acm)TAA-$NHNH_2$, was synthesized by Solid Phase Peptide Synthesis (SPPS). The Acetamidomethyl (Acm) group was used to protect the Cys2 and Cys12 at Fc-III peptide from desulfurization. The 2-Cl-(Trt)-Cl resin (625 mg, 0.25 mmol) was washed (3×DMF, 3×CH_2Cl_2, 3×DMF), and followed by swollen with CH_2Cl_2/DMF (1/1, v/v) for 30 min. Then add 5% NH_2NH_2 in DMF (v/v) to the resin for hydrazination at 30℃ for 30 min. Repeat this step for once. After another washing step, add 5% MeOH in DMF (v/v) to the resin to cap the unreacted sites. Wash the resin again, then the resin can be used to couple amino acids under microwave conditions. Each coupling cycle includes Fmoc deprotection using 20% piperidine in DMF (15 s at 75°C, 50 s at 90 °C) and amino acid coupling using 4-fold excess of 0.2 M Fmoc-protected amino acid in DMF, 0.5 M DIC in DMF, and 1.0 M Oxyma in DMF (120 s at 25°C, 240 s at 50 °C for His and Cys, 1500 s at 25°C, 120 s at 75°C for Arg, 15 s at 75°C, 110 s at 90 °C for other residues).

After the amino acids elongation, the finished peptide was cleaved from the resin with a mixture of 92.5% TFA, 2.5% water, 2.5% DODT and 2.5% TIPS. After 3 h, the resin was removed by filtration and washed with neat TFA. The combined solution was concentrated under N_2 flowing. Then, the crude peptide hydrazide was obtained by precipitating in cold Et_2O and centrifugation for three times. Finally, the desired peptides were obtained after lyophilization.

The crude peptide was dissolved in H_2O/CH_3CN and purified by preparative HPLC. Purified peptides were characterized by analytical RP-HPLC and LTQ-Orbitrap Velos.

HPLC conditions: Vydac C18 TP (300 Å, 4.6 × 250 mm), 5-85% ACN (0.1% TFA) against H_2O (0.1% TFA) over 30 min. *Flow Rate:* 1 mL/min. Tr = 18.2 min.

Mass Spectra for peptide **1**: $[M+H^+]^+$ *m/z:* 1830.928, calculated for $[M+2H^+]^{2+}$ *m/z:* 915.96, found: 915.92; $[M+3H^+]^{3+}$ *m/z:* 610.98, found: 610.95 (Figure S2A).

The Fc-III-4C peptide (**1'**), C(Acm)DC(Acm)AWHLGELVWC(Acm)TC(Acm)AA-$NHNH_2$, was synthesized by solid phase peptide synthesis (SPPS) at the same procedure as we mentioned above. While the Acetamidomethyl (Acm) group was used to protect the Cys1, Cys3, Cys13 and Cys15 at Fc-III-4C peptide from desulfurization.

HPLC conditions: Vydac C18 TP (300 Å, 4.6 × 250 mm), 5-85% ACN (0.1% TFA) against H_2O (0.1% TFA) over 30 min. *Flow Rate:* 1 mL/min. Tr = 15.2 min.

Mass Spectra for peptide **1'**: $[M+H^+]^+$ *m/z:* 2180.54, calculated for $[M+2H^+]^{2+}$ *m/z:* 1090.77, found: 1090.47; $[M+3H^+]^{3+}$ *m/z:* 727.52, found: 727.32 (Figure S6A).

2. Total chemical synthesis of DCAF molecules:

Ligation of peptide 1 and peptide 2 of DCAF1.

Sequence of peptide **2** of DCAF1:
CAAMGNHELYMRRRKPDTIEVQQMKAQAREEKHQKQMERAMLENEKKKREMAEKEKEKIEREKEEAA
AAAGLFTPNLITI.

Peptide **1** (0.9 mg, 0.5 μmol) was dissolved in 0.2 mL acidified ligation buffer (aqueous solution of 6 M GnHCl and 0.2 M NaH_2PO_4, pH 3.0). The mixture was cooled in an ice-salt bath (-15 ºC) for 15 min, and 20 μl of 0.5 M $NaNO_2$ in acidified ligation buffer was added. The activation reaction system was kept in ice-salt bath with stirring for 15 min, after which 6.8 mg MPAA in 0.2 mL acidified ligation solution with 5.5 mg peptide **2** was added. The mixture was kept in ice-salt bath for 5 min under stirring, then the pH of the solution was adjusted to 6.8-7.0 at room temperature. After 12 h, 0.2 ml of 0.1 M TCEP in a ligation buffer (pH 7.0) was added to dilute the system and the reaction system was kept for 20 min under stirring. Finally, the ligation product was analyzed and purified by RP HPLC to furnish peptide **3** in 56% isolated yield (3.6 mg).

HPLC conditions: Vydac C18 TP (300 Å, 4.6 × 250 mm), 5-85% ACN (0.1% TFA) against H_2O (0.1% TFA) over 30 min. *Flow Rate:* 1 mL/min. Tr = 16.8 min for peptide **2**; Tr = 18.3 min for peptide **3**.

Mass Spectra for peptide **2**: $[M+H^+]^+$ *m/z:* 9429.81, calculated for $[M+7H^+]^{7+}$ *m/z:* 1347.97, found: 1348.13; $[M+8H^+]^{8+}$ *m/z:* 1179.60, found: 1179.49; $[M+9H^+]^{9+}$ *m/z:* 1048.65, found: 1048.55; $[M+10H^+]^{10+}$ *m/z:* 943.88, found: 943.80; $[M+11H^+]^{11+}$ *m/z:* 858.16, found: 858.09; $[M+12H^+]^{12+}$ *m/z:* 786.73, found: 786.66; $[M+13H^+]^{13+}$ *m/z:* 726.29, found: 726.30; $[M+14H^+]^{14+}$ *m/z:* 674.49, found: 674.44 (Figure S2B).
Mass Spectra for peptide **3**: $[M+H^+]^+$ *m/z:* 11227.77, calculated for $[M+7H^+]^{7+}$ *m/z:* 1604.83, found: 1604.62; $[M+8H^+]^{8+}$ *m/z:* 1404.34, found: 1404.35; $[M+9H^+]^{9+}$ *m/z:* 1248.42, found: 1248.47; $[M+10H^+]^{10+}$ *m/z:* 1123.68, found: 1123.75; $[M+11H^+]^{11+}$ *m/z:* 1021.62, found: 1021.61; $[M+12H^+]^{12+}$ *m/z:* 936.66, found: 936.56; $[M+13H^+]^{13+}$ *m/z:* 864.60, found: 864.70; $[M+14H^+]^{14+}$ *m/z:* 802.91, found: 803.02; $[M+15H^+]^{15+}$ *m/z:* 749.45, found: 749.52 (Figure S2D).

Desulfurization of peptide 3 of DCAF1
Peptide **3** (3.4 mg, 0.3 μmol) was dissolved in 50 μl phosphate neutral buffer (pH 7.0) containing 6.0 M Gn·HCl, 0.2 M NaH_2PO_4. To the above solution, 50 μl of 1 M TCEP, 10 μl ^tBuSH and 5 μl VA-044 solution (0.1 M in phosphate neutral buffer) was added. The final pH of the solution was adjusted to 6.9 and kept at 37 ºC with stirring about 5 hours. Finally, the desulfurization product was analyzed and purified by RP HPLC to furnish peptide **4** in 41% isolated yield (1.48 mg).

HPLC conditions: Vydac C18 TP (300 Å, 4.6 × 250 mm), 5-85% ACN (0.1% TFA) against H_2O (0.1% TFA) over 30 min. *Flow Rate:* 1 mL/min. Tr = 18.5 min.

Mass Spectra for peptide **4**: $[M+H^+]^+$ *m/z:* 11195.71, calculated for $[M+7H^+]^{7+}$ *m/z:* 1600.25, found: 1600.18; $[M+8H^+]^{8+}$ *m/z:* 1400.34, found: 1400.25; $[M+9H^+]^{9+}$ *m/z:* 1244.86, found: 1244.80; $[M+10H^+]^{10+}$ *m/z:* 1120.47, found: 1120.44; $[M+11H^+]^{11+}$ *m/z:* 1018.70, found: 1018.78 (Figure S2E).

Remove of ACM in peptide 4 of DCAF1.
Peptide **4** (1.35 mg, 0.11 μmol) was dissolved in 200 μl of 32 mM AgOAc(in 50% CH_3COOH), and the

reaction was stirred at r.t. for 4 hours. Subsequently, 1-3 μl of 1M Dithiothreitol (DTT) dissolved in 0.2 M phosphate solution containing 6 M Gn·HCl (pH 7.0) was added to convert the silver thiolates on proteins to free thiols. After stirred violently, the supernatant was purified and analyzed by RP-HPLC to furnish peptide **5** in 30% isolated yield (0.45 mg).

HPLC conditions: Vydac C18 TP (300 Å, 4.6 × 250 mm), 5-85% ACN (0.1% TFA) against H_2O (0.1% TFA) over 30 min. *Flow Rate:* 1 mL/min. Tr = 18.9 min.

Mass Spectra for peptide **4**: $[M+H^+]^+$ *m/z:* 11053.72, calculated for $[M+7H^+]^{7+}$ *m/z:* 1579.96, found: 1579.77; $[M+8H^+]^{8+}$ *m/z:* 1382.59, found: 1382.49; $[M+9H^+]^{9+}$ *m/z:* 1229.08, found: 1229.13; $[M+10H^+]^{10+}$ *m/z:* 1106.27, found: 1106.23; $[M+11H^+]^{11+}$ *m/z:* 1005.79, found: 1005.77; $[M+12H^+]^{12+}$ *m/z:* 922.06, found: 922.06; $[M+13H^+]^{13+}$ *m/z:* 851.21, found: 851.23; $[M+14H^+]^{14+}$ *m/z:* 790.48, found: 790.51; $[M+15H^+]^{15+}$ *m/z:* 737.85, found: 737.77 (Figure 2C).

Ligation of peptide 1 and peptide 2 of DCAF2

Sequence of peptide **2** of DCAF2:
CAAMGNHELYMRRRKPDTIEVQQMKAQAREEKHQKQMERAMLENEKKKREMAEKEKEKIEREKEEAA AAARMAILGDTAWDFGSLA.

The ligation method is same with mentioned above. In brief, peptide **1** (1.5mg, 0.8 μmol) was dissolved in 0.2 mL acidified ligation buffer. The mixture was cooled at -15 ºC for 15min, and add 20 μl of 0.5 M $NaNO_2$. After another -15 ºC for 15 min, 6.8 mg MPAA with 5 mg peptide **2** in 0.2 mL acidified ligation solution was added. The mixture was adjusted the pH to 6.8-7.0 at room temperature. After 12 h, 0.2 ml of 0.1 M TCEP was added to dilute the system and the reaction system was kept for 20 min under stirring. Finally, the ligation product was analyzed and purified by RP HPLC to furnish peptide **3** in 39% isolated yield (2.3 mg).

HPLC conditions: Vydac C18 TP (300 Å, 4.6 × 250 mm), 5-85% ACN (0.1% TFA) against H_2O (0.1% TFA) over 30 min. *Flow Rate:* 1 mL/min. Tr = 17.6 min for peptide **2**; Tr = 18.5 min for peptide **3**.

Mass Spectra for peptide **2**: $[M+H^+]^+$ *m/z:* 10065.02, calculated for $[M+8H^+]^{8+}$ *m/z:* 1259.00, found: 1258.98; $[M+9H^+]^{9+}$ *m/z:* 1119.22, found: 1119.22; $[M+10H^+]^{10+}$ *m/z:* 1007.40, found: 1007.32; $[M+11H^+]^{11+}$ *m/z:* 915.91, found: 915.85; $[M+12H^+]^{12+}$ *m/z:* 839.67, found: 839.72; $[M+13H^+]^{13+}$ *m/z:* 775.16, found: 775.14; $[M+14H^+]^{14+}$ *m/z:* 719.86, found: 719.93 (Figure S4A);
Mass Spectra for peptide **3**: $[M+H^+]^+$ *m/z:* 11863.42, calculated for $[M+8H^+]^{8+}$ *m/z:* 1483.80, found: 1483.80 $[M+9H^+]^{9+}$ *m/z:* 1319.05, found: 1319.05; $[M+10H^+]^{10+}$ *m/z:* 1187.24, found: 1187.25; $[M+11H^+]^{11+}$ *m/z:* 1079.40, found: 1079.41; $[M+12H^+]^{12+}$ *m/z:* 989.54, found: 989.54; $[M+13H^+]^{13+}$ *m/z:* 913.49, found: 913.50 (Figure S4B);

Desulfurization of peptide 3 of DCAF2

The method is same with mentioned above. In brief, peptide **3** (2.3mg, 0.5 μmol) was dissolved in 50 μl phosphate neutral buffer (pH 7.0). To the above solution, 50 μl of 1 M TCEP, 10 μl ^tBuSH and 5 μl VA-044 solution was added. The final pH of the solution was adjusted to 6.9 and kept at 37 ºC with stirring about 5 hours. Finally, the desulfurization product was analyzed and purified by RP HPLC to furnish peptide **4** in 25% isolated yield (0.87 mg).

HPLC conditions: Vydac C18 TP (300 Å, 4.6 × 250 mm), 5-85% ACN (0.1% TFA) against H_2O (0.1% TFA) over 30 min. *Flow Rate:* 1 mL/min. Tr = 20.1 min.

Mass Spectra for peptide **4**: $[M+H^+]^+$ *m/z:* 11831.36, calculated for $[M+7H^+]^{7+}$ *m/z:* 1691.05, found: 1691.08; $[M+8H^+]^{8+}$ *m/z:* 1479.80, found: 1479.82; $[M+9H^+]^{9+}$ *m/z:* 1315.48, found: 1315.50; $[M+10H^+]^{10+}$ *m/z:* 1184.04, found: 1184.05; $[M+11H^+]^{11+}$ *m/z:* 1076.49, found: 1076.50; $[M+12H^+]^{12+}$ *m/z:* 986.86, found: 986.87; $[M+13H^+]^{13+}$ *m/z:* 911.03, found: 911.04 (Figure S4C);

Remove of ACM in peptide 4 of DCAF2.

The method is same with mentioned above. In brief, peptide **4** (0.87 mg, 0.07 μmol) was dissolved in 200 μl of 32 mM AgOAc (in 50% CH_3COOH), and the reaction was stirred at r.t. for 4 hours. Subsequently, 1-3 μl of 1M Dithiothreitol (DTT) was added. After stirred violently, the supernatant was purified and analyzed by RP-HPLC to furnish peptide **5** in 28% isolated yield (0.24 mg).

HPLC conditions: Vydac C18 TP (300 Å, 4.6 × 250 mm), 5-85% ACN (0.1% TFA) against H_2O (0.1% TFA) over 30 min. *Flow Rate:* 1 mL/min. Tr = 20.4 min.

Mass Spectra for peptide **5**: $[M+H^+]^+$ *m/z:* 11689.37, calculated for $[M+8H^+]^{8+}$ *m/z:* 1462.05, found: 1461.92; $[M+9H^+]^{9+}$ *m/z:* 1299.71, found: 1299.59; $[M+10H^+]^{10+}$ *m/z:* 1169.84, found: 1169.86; $[M+11H^+]^{11+}$ *m/z:* 1063.58, found: 1063.61; $[M+12H^+]^{12+}$ *m/z:* 975.03, found: 975.07; $[M+13H^+]^{13+}$ *m/z:* 900.11, found: 900.16; $[M+14H^+]^{14+}$ *m/z:* 835.88, found: 835.95; $[M+15H^+]^{15+}$ *m/z:* 780.22, found: 780.24; $[M+16H^+]^{16+}$ *m/z:* 731.52, found: 731.54; $[M+17H^+]^{17+}$ *m/z:* 688.55, found: 688.64 (Figure S4D).

Ligation of peptide 1 and peptide 2 of DCAF3

Sequence of peptide **2** of DCAF3:
CAAMGNHELYMRRRKPDTIEVQQMKAQAREEKHQKQMERAMLENEKKKREMAEKEKEKIEREKEEAA
AAAVDRGWGNGCGLFA.

The ligation method is same with mentioned above. In brief, peptide **1** (0.8mg, 0.4 μmol) was dissolved in 0.2 mL acidified ligation buffer. The mixture was cooled at -15 ºC for 15min, and add 20 μl of 0.5 M $NaNO_2$. After another -15 ºC for 15 min, 6.8 mg MPAA with 3.5 mg peptide **2** in 0.2 mL acidified ligation solution was added. The mixture was adjusted the pH to 6.8-7.0 at room temperature. After 12 h, 0.2 ml of 0.1 M TCEP was added to dilute the system and the reaction system was kept for 20 min under stirring. Finally, the ligation product was analyzed and purified by RP HPLC to furnish peptide **3** in 31% isolated yield (1.3 mg).

HPLC conditions: Vydac C18 TP (300 Å, 4.6 × 250 mm), 5-85% ACN (0.1% TFA) against H_2O (0.1% TFA) over 30 min. *Flow Rate:* 1 mL/min. Tr = 15.5 min for peptide **2**; Tr = 18.2 min for peptide **3**.

Mass Spectra for peptide **2**: $[M+H^+]^+$ *m/z:* 9692.08, calculated for $[M+7H^+]^{7+}$ *m/z:* 1385.44, found: 1385.42; $[M+8H^+]^{8+}$ *m/z:* 1212.39, found: 1212.38; $[M+9H^+]^{9+}$ *m/z:* 1077.79, found: 1077.78; $[M+10H^+]^{10+}$ *m/z:* 970.11, found: 970.10 (Figure S5A);

Mass Spectra for peptide **3**: $[M+H^+]^+$ *m/z:* 11490.96, calculated for $[M+8H^+]^{8+}$ *m/z:* 1437.25, found: 1437.30; $[M+9H^+]^{9+}$ *m/z:* 1277.66, found: 1277.71; $[M+10H^+]^{10+}$ *m/z:* 1150.00, found: 1150.03; $[M+11H^+]^{11+}$ *m/z:* 1045.54, found: 1045.58; $[M+12H^+]^{12+}$ *m/z:* 958.50, found: 958.53; $[M+13H^+]^{13+}$ *m/z:*

884.84, found: 884.88; $[M+14H^+]^{14+}$ *m/z:* 821.71, found: 821.82; $[M+15H^+]^{15+}$ *m/z:* 767.00, found: 767.03 (Figure S5B);

Remove of ACM in peptide 3 of DCAF3.

The method is same with mentioned above. In brief, peptide **3** (1.3 mg, 0.11 μmol) was dissolved in 200 μl of 32 mM AgOAc (in 50% CH3COOH), and the reaction was stirred at r.t. for 4 hours. Subsequently, 1-3 μl of 1M Dithiothreitol (DTT) was added. After stirred violently, the supernatant was purified and analyzed by RP-HPLC to furnish peptide **5** in 33% isolated yield (0.4 mg).

HPLC conditions: Vydac C18 TP (300 Å, 4.6 × 250 mm), 5-85% ACN (0.1% TFA) against H_2O (0.1% TFA) over 30 min. *Flow Rate:* 1 mL/min. Tr = 18.6 min.

Mass Spectra for peptide **5**: $[M+H^+]^+$ *m/z:* 11348.97, calculated for $[M+8H^+]^{8+}$ *m/z:* 1419.50, found: 1419.50; $[M+9H^+]^{9+}$ *m/z:* 1261.89, found: 1261.87; $[M+10H^+]^{10+}$ *m/z:* 1135.80, found: 1135.79; $[M+11H^+]^{11+}$ *m/z:* 1032.63, found: 1032.61; $[M+12H^+]^{12+}$ *m/z:* 946.66, found: 946.65; $[M+13H^+]^{13+}$ *m/z:* 873.92, found: 873.91; $[M+14H^+]^{14+}$ *m/z:* 811.57, found: 811.55 (Figure S5C).

Ligation of peptide 1' and peptide 2 of DCAF4.

Sequence of peptide **2** of DCAF4:
CAAMGNHELYMRRRKPDTIEVQQMKAQAREEKHQKQMERAMLENEKKKREMAEKEKEKIEREKEEAA AAASEHETRLVANLLGGGSLRWNPADYGGIKKIRGSLDYTGK.

Peptide **1'** (1.2 mg, 0.5 μmol) was dissolved in 0.2 mL acidified ligation buffer (aqueous solution of 6 M GnHCl and 0.2 M NaH_2PO_4, pH 3.0). The mixture was cooled in an ice-salt bath (-15 ºC) for 15 min, and 20 μl of 0.5 M $NaNO_2$ in acidified ligation buffer was added. The activation reaction system was kept in ice-salt bath with stirring for 15 min, after which 6.8 mg MPAA in 0.2 mL acidified ligation solution with 4.5 mg peptide **2** was added. The mixture was kept in ice-salt bath for 5 min under stirring, then the pH of the solution was adjusted to 6.8-7.0 at room temperature. After 12 h, 0.2 ml of 0.1 M TCEP in a ligation buffer (pH 7.0) was added to dilute the system and the reaction system was kept for 20 min under stirring. Finally, the ligation product was analyzed and purified by RP HPLC to furnish peptide **3** in 31 % isolated yield (1.4 mg).

HPLC conditions: Vydac C18 TP (300 Å, 4.6 × 250 mm), 5-85% ACN (0.1% TFA) against H_2O (0.1% TFA) over 30 min. *Flow Rate:* 1 mL/min. Tr = 18.4 min for peptide **2**; Tr = 18.5 min for peptide **3**.

Mass Spectra for peptide **2**: $[M+H^+]^+$ *m/z:* 12572.34, calculated for $[M+9H^+]^{9+}$ *m/z:* 1397.82, found: 1397.79; $[M+10H^+]^{10+}$ *m/z:* 1258.14, found: 1258.12; $[M+11H^+]^{11+}$ *m/z:* 1143.86, found: 1143.83; $[M+12H^+]^{12+}$ *m/z:* 1048.62, found: 1048.68; $[M+13H^+]^{13+}$ *m/z:* 968.03, found: 968.02; $[M+14H^+]^{14+}$ *m/z:* 898.96, found: 898.95; $[M+15H^+]^{15+}$ *m/z:* 839.10, found: 839.15; $[M+16H^+]^{16+}$ *m/z:* 786.72, found: 786.70; $[M+17H^+]^{17+}$ *m/z:* 740.50, found: 740.54; $[M+18H^+]^{18+}$ *m/z:* 699.42, found: 699.40; $[M+19H^+]^{19+}$ *m/z:* 662.66, found: 662.69; $[M+20H^+]^{20+}$ *m/z:* 629.57, found: 629.61 (Figure S6B).
Mass Spectra for peptide **3**: $[M+H^+]^+$ *m/z:* 14719.82, calculated for $[M+11H^+]^{11+}$ *m/z:* 1339.08, found: 1339.04; $[M+12H^+]^{12+}$ *m/z:* 1227.58, found: 1227.53; $[M+13H^+]^{13+}$ *m/z:* 1133.22, found:1133.17; $[M+14H^+]^{14+}$ *m/z:* 1052.35, found: 1052.31; $[M+15H^+]^{15+}$ *m/z:* 982.26, found: 982.22; $[M+16H^+]^{16+}$ *m/z:* 920.93, found: 920.83; $[M+17H^+]^{17+}$ *m/z:* 866.82, found: 866.85; $[M+18H^+]^{18+}$ *m/z:* 818.72, found: 818.63;

$[M+19H^+]^{19+}$ *m/z:* 775.68, found: 775.65; $[M+20H^+]^{20+}$ *m/z:* 736.95, found: 736.87 (Figure S6C).

Desulfurization of peptide 3 of DCAF4

Peptide **3** (0.67 mg, 0.04 μmol) was dissolved in 50 μl phosphate neutral buffer (pH 7.0) containing 6.0 M Gn·HCl, 0.2 M NaH_2PO_4. To the above solution, 50 μl of 1 M TCEP, 10 μl ^tBuSH and 5 μl VA-044 solution (0.1 M in phosphate neutral buffer) was added. The final pH of the solution was adjusted to 6.9 and kept at 37 ºC with stirring about 4 hours. Finally, the desulfurization product was analyzed and purified by RP HPLC to furnish peptide **4** in 75% isolated yield (0.5 mg).

HPLC conditions: Vydac C18 TP (300 Å, 4.6 × 250 mm), 20-45% ACN (0.1% TFA) against H_2O (0.1% TFA) over 20 min. *Flow Rate:* 1 mL/min. Tr = 16.5 min.

Mass Spectra for peptide **4**: $[M+H^+]^+$ *m/z:* 14687.76, calculated for $[M+11H^+]^{11+}$ *m/z:* 1336.17, found: 1336.13; $[M+12H^+]^{12+}$ *m/z:* 1224.90, found: 1224.79; $[M+13H^+]^{13+}$ *m/z:* 1130.76, found:113065; $[M+14H^+]^{14+}$ *m/z:* 1050.06, found: 1050.06; $[M+15H^+]^{15+}$ *m/z:* 980.12, found: 980.10; $[M+16H^+]^{16+}$ *m/z:* 918.93, found: 918.90; $[M+17H^+]^{17+}$ *m/z:* 864.93, found: 864.97; $[M+18H^+]^{18+}$ *m/z:* 816.94, found: 816.92; $[M+19H^+]^{19+}$ *m/z:* 774.00, found: 773.92 (Figure S6D).

Remove of ACM in peptide 4 of DCAF4.

Peptide **4** (0.5 mg, 0.03 μmol) was dissolved in 200 μl of 32 mM AgOAc(in 50% CH_3COOH), and the reaction was stirred at r.t. for 4 hours. Subsequently, 1-3 μl of 1M Dithiothreitol (DTT) dissolved in 0.2 M phosphate solution containing 6 M Gn·HCl (pH 7.0) was added to convert the silver thiolates on proteins to free thiols. After stirred violently, the supernatant was purified and analyzed by RP-HPLC to furnish peptide **5** in 24% isolated yield (0.12 mg).

HPLC conditions: Vydac C18 TP (300 Å, 4.6 × 250 mm), 5-85% ACN (0.1% TFA) against H_2O (0.1% TFA) over 30 min. *Flow Rate:* 1 mL/min. Tr = 20.0 min.

Mass Spectra for peptide **5**: $[M+H^+]^+$ *m/z:* 14399.41, calculated for $[M+11H^+]^{11+}$ *m/z:* 1309.95, found: 1309.75; $[M+12H^+]^{12+}$ *m/z:* 1200.88, found: 1200.85; $[M+13H^+]^{13+}$ *m/z:* 1108.58, found:1108.56; $[M+14H^+]^{14+}$ *m/z:* 1029.47, found: 1029.45; $[M+15H^+]^{15+}$ *m/z:* 960.90, found: 960.82; $[M+16H^+]^{16+}$ *m/z:* 900.91, found: 9900.83; $[M+17H^+]^{17+}$ *m/z:* 847.97, found: 847.96; $[M+18H^+]^{18+}$ *m/z:* 800.92, found: 800.85; $[M+19H^+]^{19+}$ *m/z:* 758.82, found: 758.75 (Figure S6E).

Supporting Figures

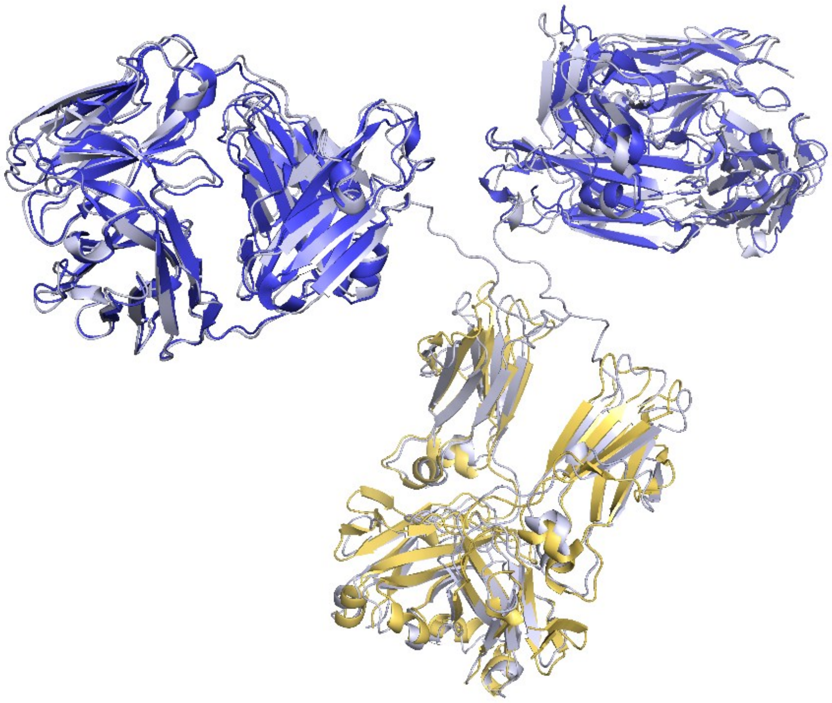

Figure S1. Superposition of the crystal structures of 2H2 Fab (4KVC) (blue) and Fc region binding to Fc-III peptide complex (1DN2) (yellow) with the whole IgG molecule (1IGT) (gray).

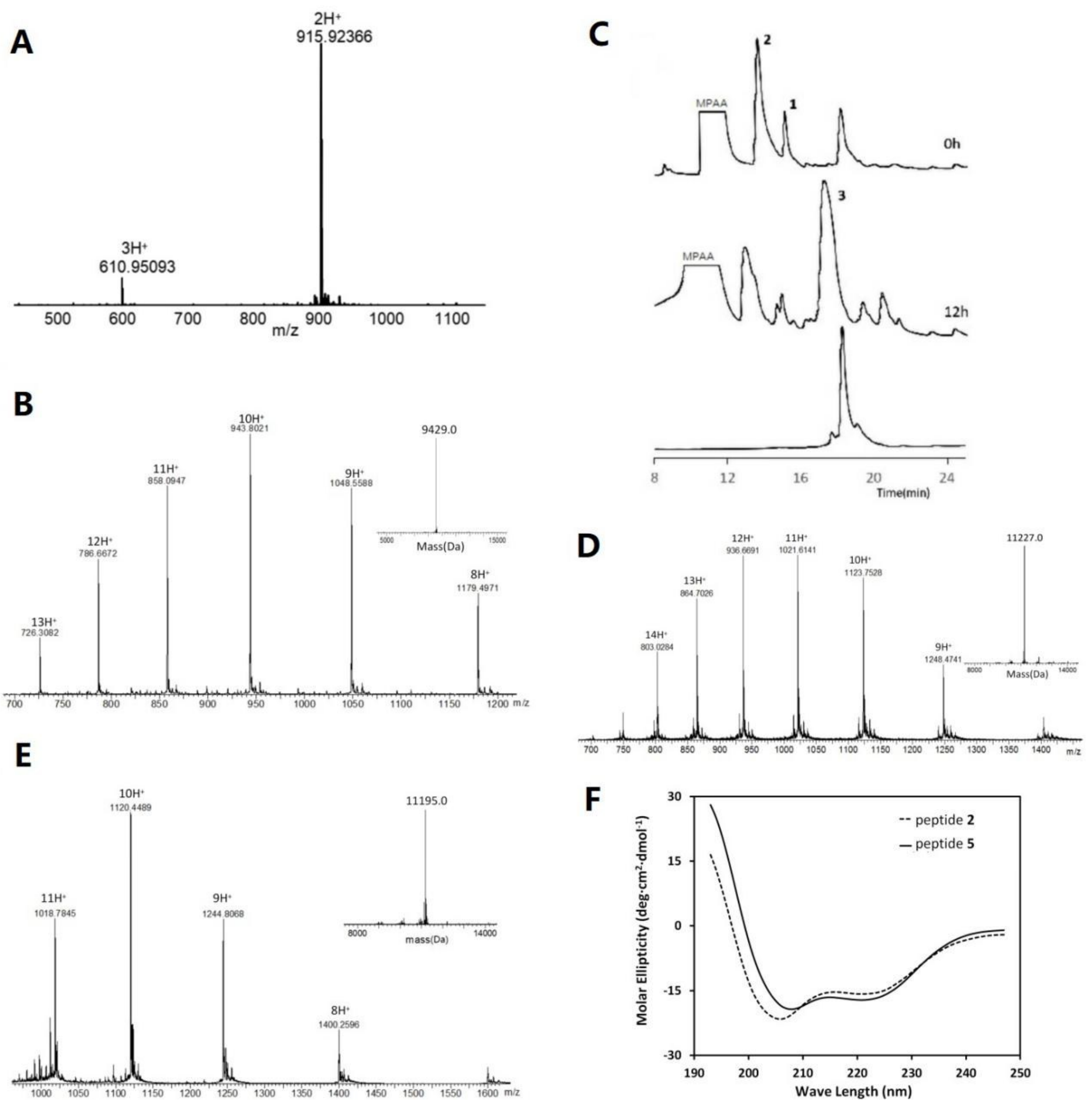

Figure S2. Characterization of all the intermediates of DCAF1 molecules and circular dichroism spectra. (A) Peptide **1** with hydrazine and Acm protection detected by LTQ-Orbitrap Velos LC-MS (Thermo), indicating that the monoisotopic molecular weight in single charge is 1830.84. (B) The products of DCAF1-peptide **2** is detected by Synapt G2 Si (Waters) LC-MS, the ESI-MS spectrum gave an observed mass of 9429.0 Da (calcd 9429.8 Da, average isotopes). (C) Auxiliary-assisted ligation monitored by RP-HPLC, 0 h (top), 12 h (middle) and purified peptide **3** (bottom). (D&E) The products of DCAF1-peptide **3**, **4** is detected by Synapt G2 Si (Waters) LC-MS, the ESI-MS spectrum gave an observed mass of 11227.0 Da (calcd 11227.7 Da, average isotopes) and 11195.0 Da (calcd 11195.7 Da, average isotopes), respectively. (F) Circular dichroism spectra of peptide **2** and peptide **5** in DCAF1. The final concentrations of peptide **2** and peptide **5** were all 10 μM. The spectrum for each peptide was performed in triplicates, averaged, subtracted from blank and smoothed.

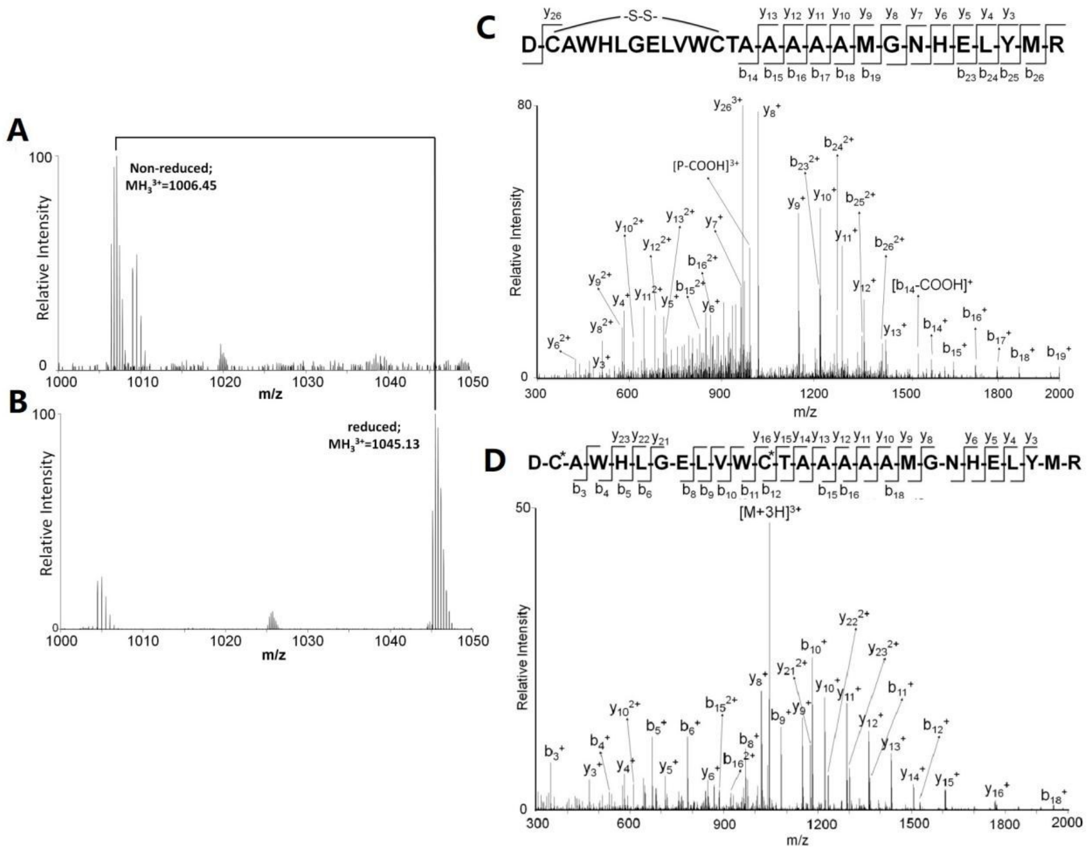

Figure S3. MS spectra of non-reducing and reducing tryptic peptides containing Fc-III in DCAF1. (A) The mono-isotope peak at m/z 1006.45 matched the triply charged peptide DCAWHLGELVWCTAAAAAMGNHELYMR where Cys2 and Cys12 form a disulfide bond. (B) The mono-isotope peak at m/z 1045.13 matched the triply charged peptide of (A) after reduction and alkylation. (C) The MS/MS spectrum of (A); the labeled peaks correspond to masses of y ions and b ions of the selected peptide. (D) The MS/MS spectrum of (B); the labeled peaks correspond to masses of y ions and b ions of the selected peptide. while * representitive the carbamidomethyl modification on Cys residues.

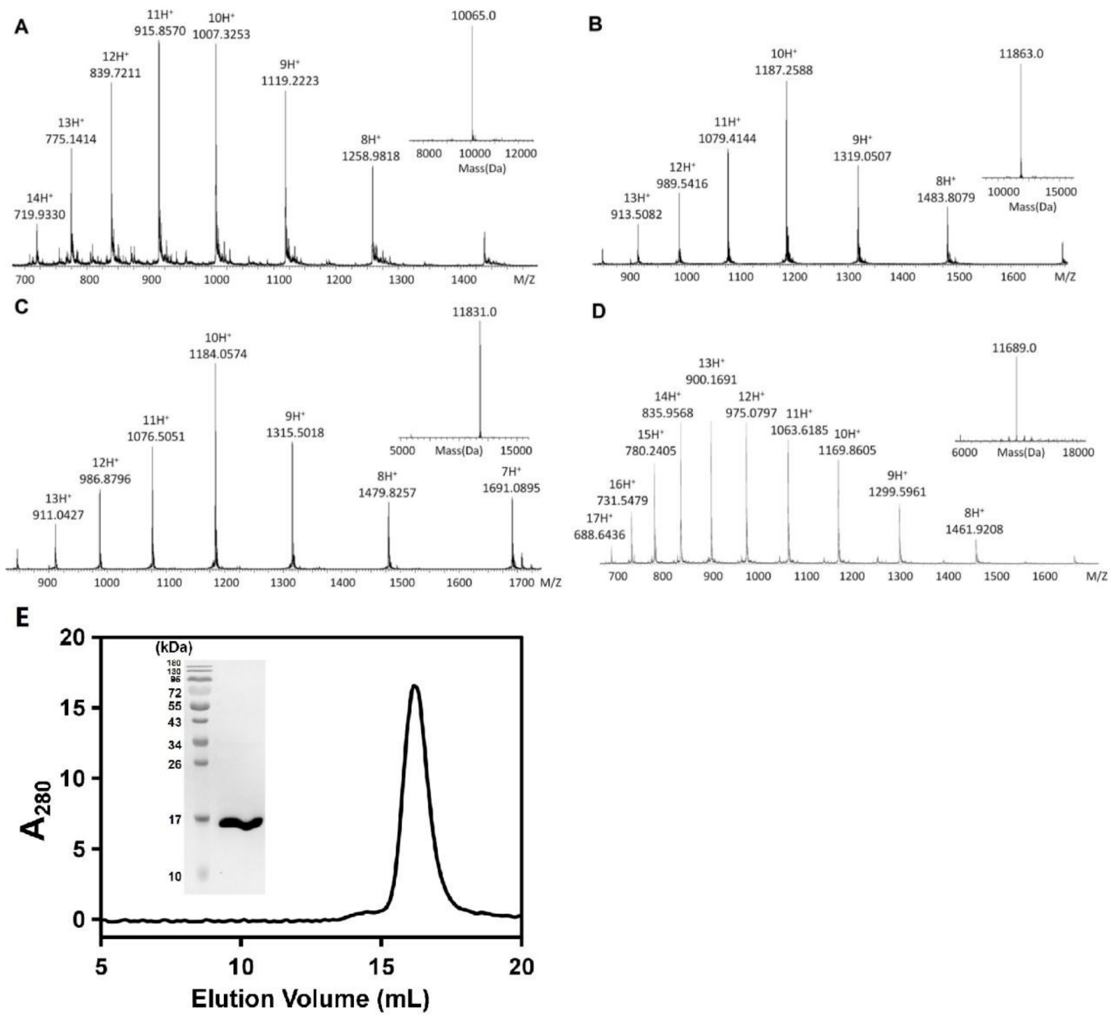

Figure S4. Characterization of all the intermediates of DCAF2 molecules. (A) The ESI-MS spectrum of the product of DCAF2-peptide **2** gave an observed mass of 10065.0 Da (calcd 10064.5 Da, average isotopes). (B) The ESI-MS spectrum of the product of DCAF2-peptide **3** gave an observed mass of 11863.0 Da (calcd 11863.4 Da, average isotopes). (C) The ESI-MS spectrum of the product of DCAF2-peptide **4** gave an observed mass of 11831.0.0 Da (calcd 11831.3 Da, average isotopes). (D) The ESI-MS spectrum of the product of DCAF2-peptide **5** gave an observed mass of 11689.0 Da (calcd 11689.3 Da, average isotopes). All the products are detected by Synapt G2 Si (Waters) LC-MS. (E) Characterization of purified DCAF2-peptide **5** by SEC and SDS-PAGE (left, molecular weight ladder; right, peptide **5**), the calculated final purity of DCAF2 is 97.8%.

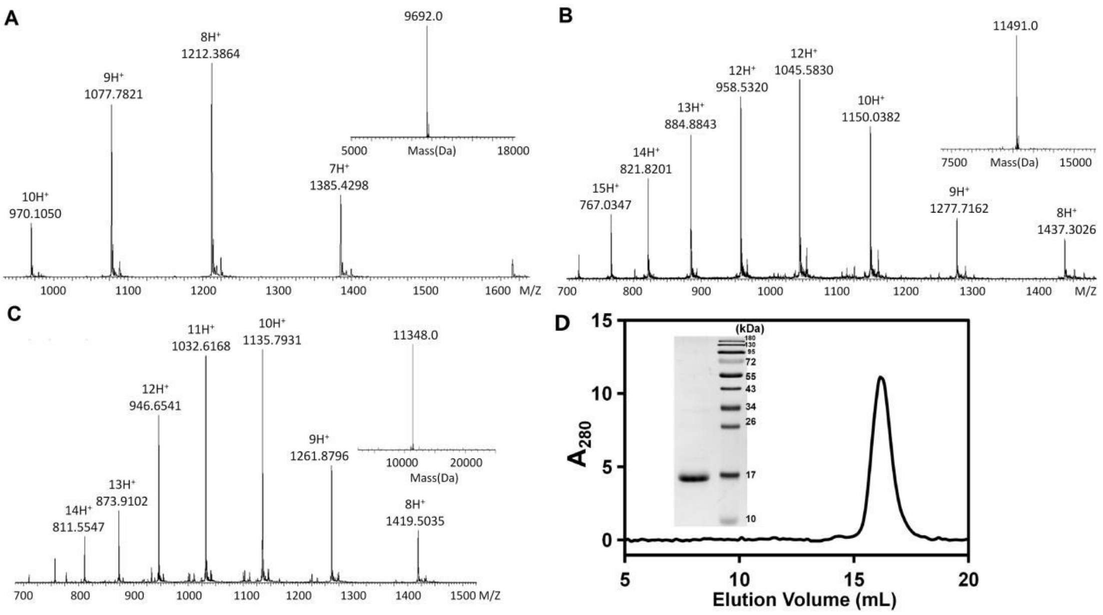

Figure S5. Characterization of all the intermediates of DCAF3 molecules. (A) The ESI-MS spectrum of the product of DCAF3-peptide **2** gave an observed mass of 9692.0 Da (calcd 9692.1 Da, average isotopes). (B) The ESI-MS spectrum of the product of DCAF3-peptide **3** gave an observed mass of 11491.0 Da (calcd 11490.9 Da, average isotopes). (C) The ESI-MS spectrum of the product of DCAF3-peptide **5** gave an observed mass of 11348.0 Da (calcd 11358.9 Da, average isotopes). All the products are detected by Synapt G2 Si (Waters) LC-MS. (D) Characterization of purified DCAF3-peptide **5** by SEC and SDS-PAGE (left, peptide **5**; right, molecular weight ladder), the calculated final purity of DCAF4 is 97.7%.

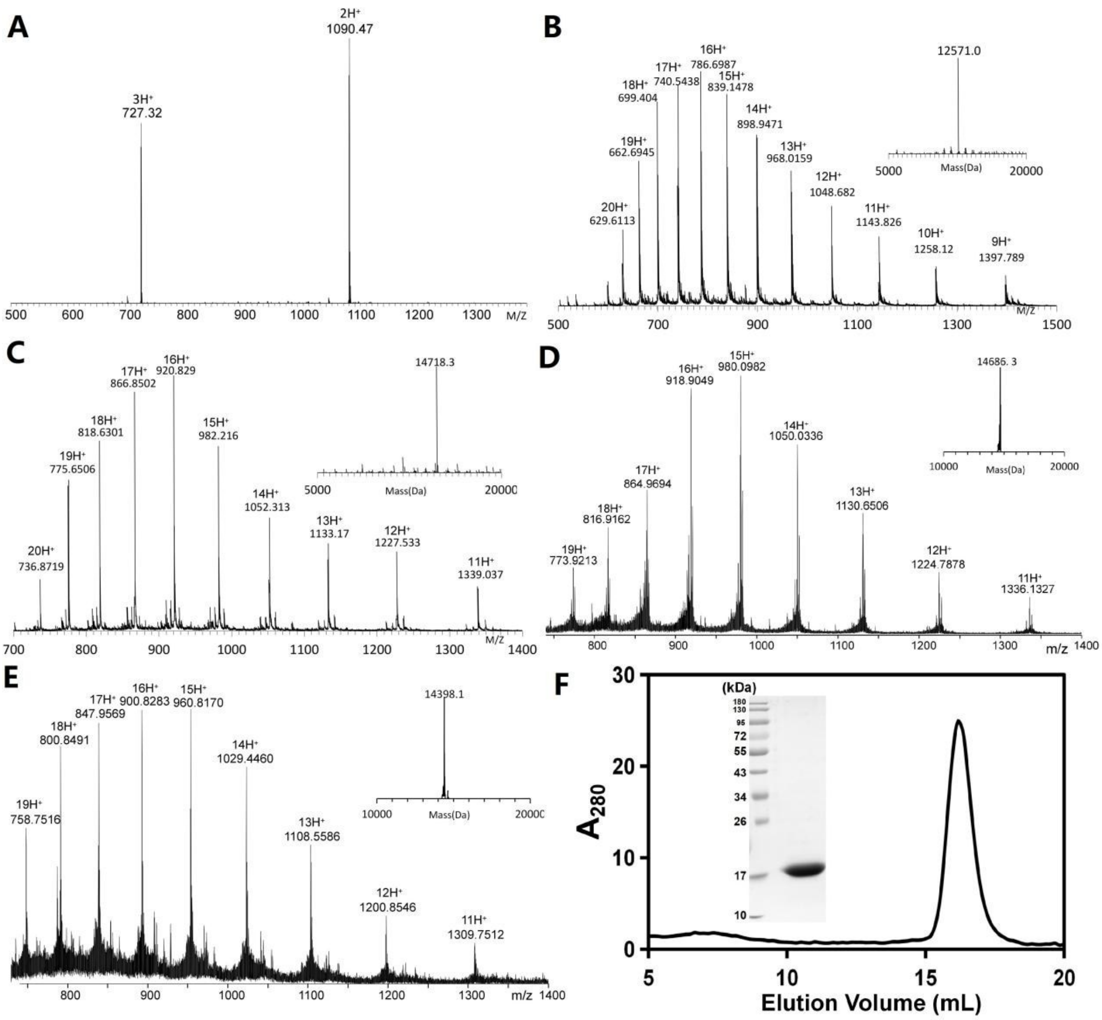

Figure S6. Characterization of all the intermediates of DCAF4 molecules. (A) The product of DCAF4-peptide **1** is detected by LTQ-Orbitrap Velos LC-MS (Thermo), indicating the monoisotopic molecular weight in single charge is 2179.93. (B&C) The products of DCAF4-peptide **2**, **3** is detected by Synapt G2 Si (Waters) LC-MS, the ESI-MS spectrum gave an observed mass of 12571.0 Da (calcd 12571.3 Da, average isotopes) and 14718.0 Da (calcd 14718.8 Da, average isotopes), respectively. (D&E) The products of DCAF4-peptide **4**, **5** is detected by LTQ-Orbitrap Velos LC-MS (Thermo), the ESI-MS spectrum gave an observed mass of 14686.0 Da (calcd 14686.7 Da, average isotopes) and 14398.0 Da (calcd 14398.4 Da, average isotopes), respectively. (F) Characterization of purified DCAF4-peptide **5** by SEC and SDS-PAGE (left, molecular weight ladder; right, peptide **5**), the calculated final purity of DCAF4 is 97.1%.

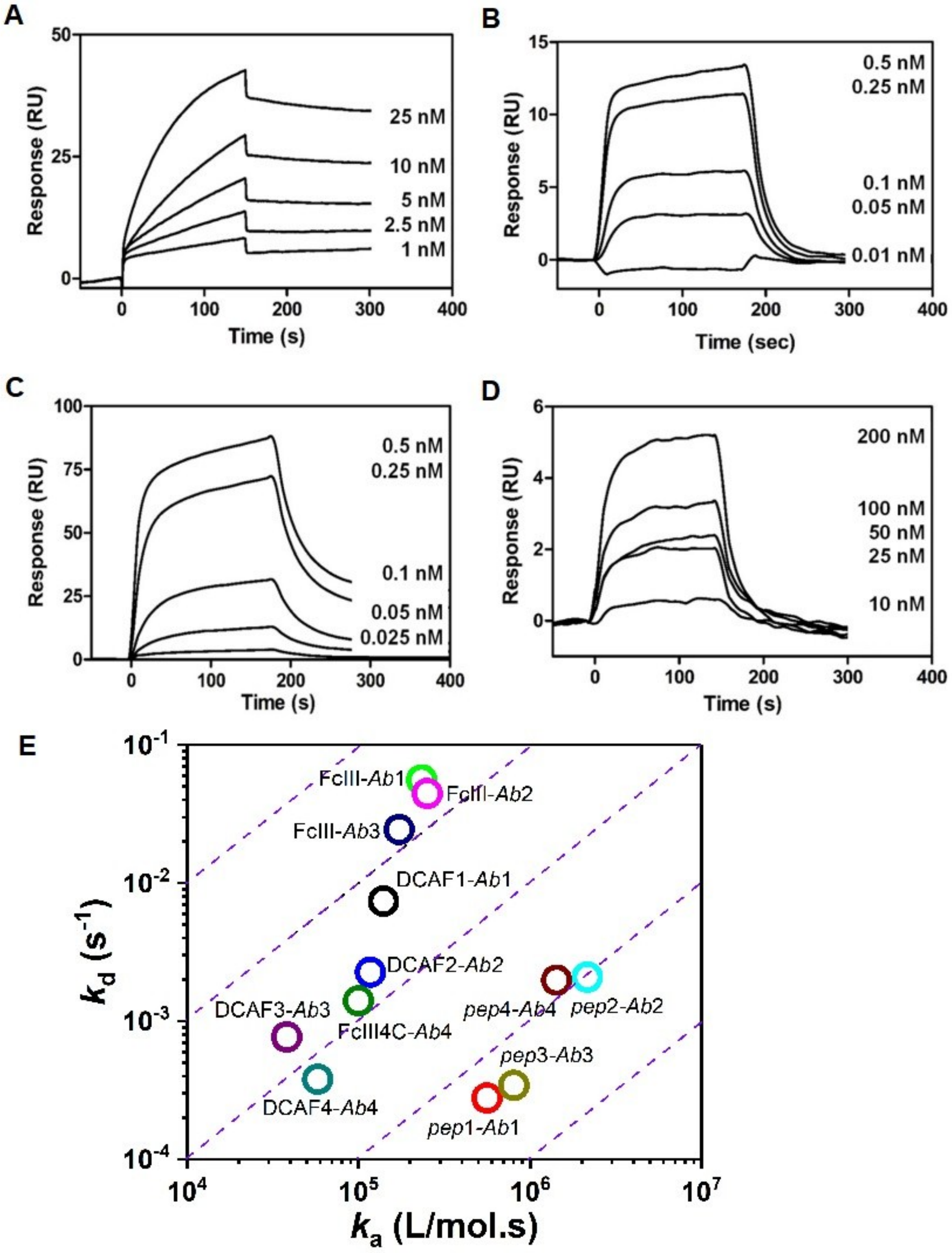

Figure S7. SPR analysis of the binding affinity between different ligands and *Ab*1. (A&B) SPR result showed the K_d value between *Ab*1 and *pep*1 or Fc-III was 0.50 nM and 0.24 μM, respectively. (C&D) SPR result showed the K_d value between *Ab*1 and DCAF1 or DCAF2 was 52.9 nM and 0.28 μM, respectively. (E) Summary of the kinetic constants of antigenic peptides, Fc-III mimetics and DCAF molecules binding to their cognate antibodies measured by SPR. The horizontal axis indicates binding rate constants (k_a), while the vertical axis shows dissociation rate constants (k_d).

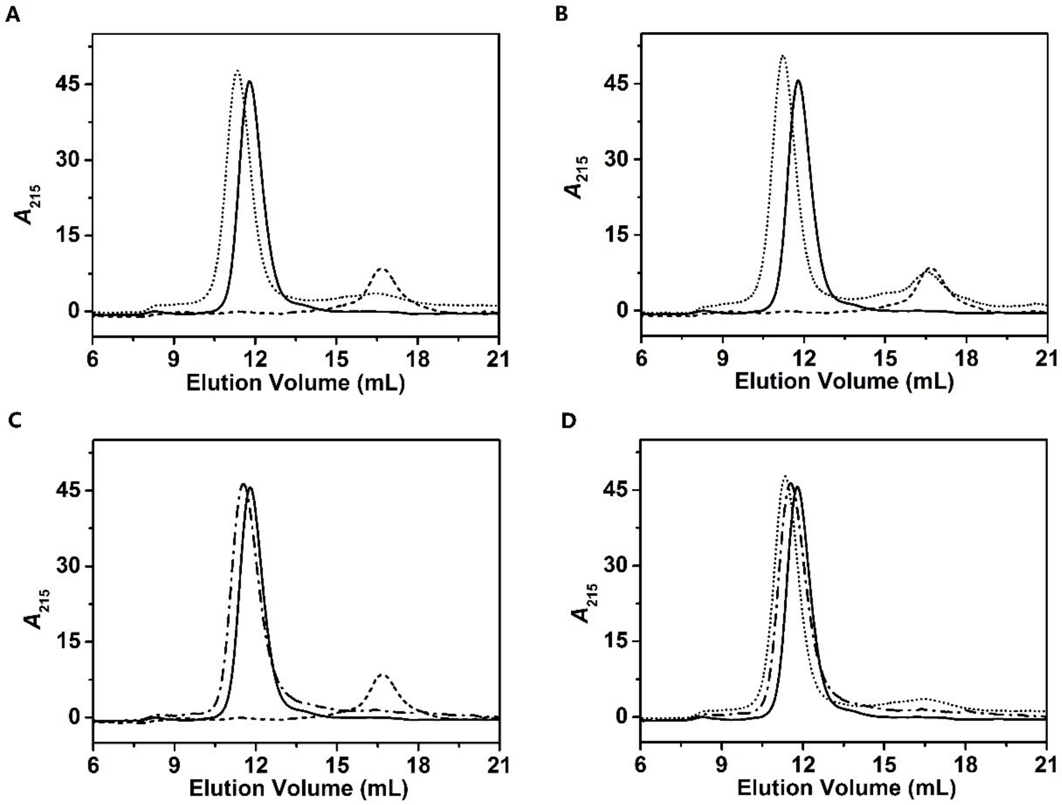

Figure S8. Stoichiometry measurement of DCAF1 with *Ab*1 by SEC. The concentration of *Ab*1 is 1 μM, while the concentrations of DCAF1 are 2 μM (A), 4 μM (B) and 1 μM (C), respectively. (D) compares the elution volume of the peaks appeared in (A) and (C). Solid, dash, dot and dash-dot lines represent *Ab*1, DCAF1, 1:2 *Ab*1 with DCAF1 complex, and the mixture of 1:2 complex, 1:1 complex and *Ab*1, respectively.

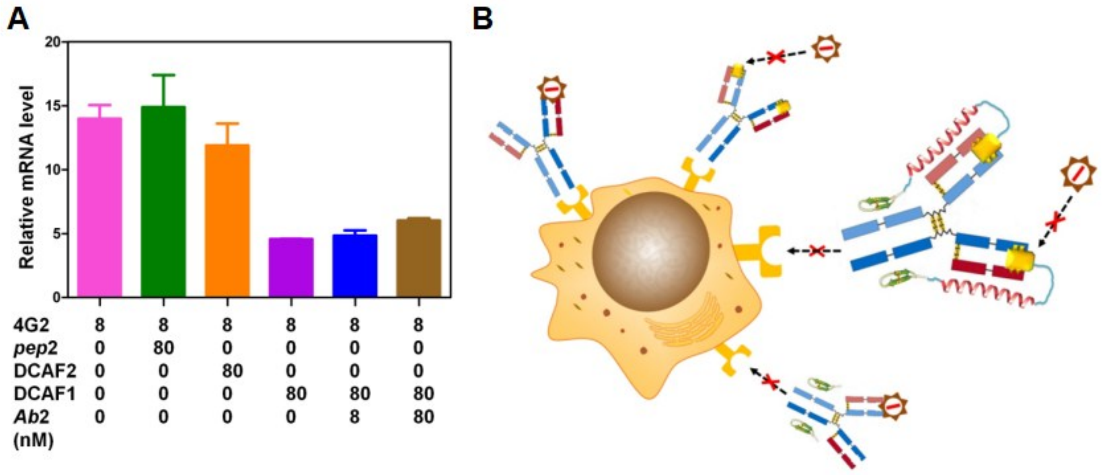

Figure S9. The competitional ADE assay and proposed mechanism of DCAF1 molecule blocking ADE effect. (A) The competitional ADE assay of 4G2 and DCAF1 using *Ab*2 as irrelevant antibody. (B) The DCAF1 molecule can inhibit antibody-virus binding and antibody-Fc receptor interaction simultaneously to block ADE effect.

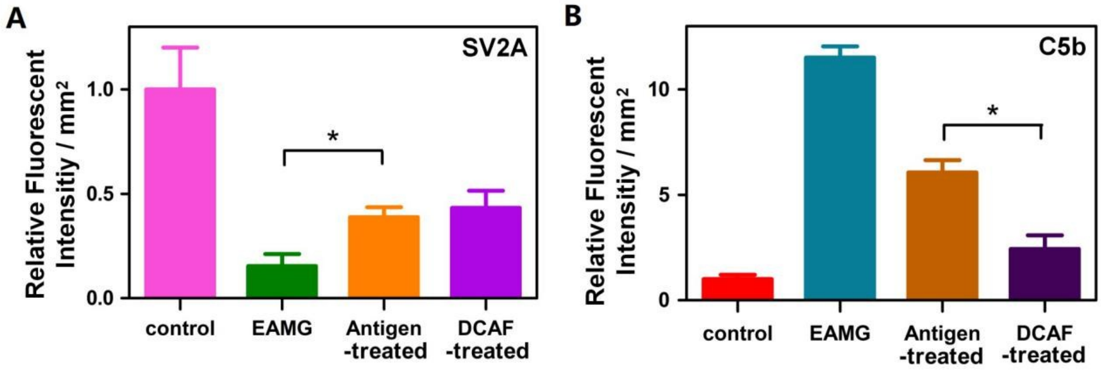

Figure S10. Relative fluorescent intensities of SV2A (A) and C5b (B) derived from immuno-histofluorescence images. Data are shown as the means ± SD from two images, where * indicates $p < 0.05$.

Table S1. Summary of the detailed composition in DCAF molecules.

	Antigen part	Antigen sequence	Fc-III mimetic
DCAF1	*pep*1	GLFTPNLITI	Fc-III
DCAF2	*pep*2	RMAILGDTAWDFGSL	Fc-III
DCAF3	*pep*3	VDRGWGNGCGL	Fc-III
DCAF4	*pep*4	SEHETRLVANLLGGGSLRWN<br>PADYGGIKKIRGSLDYTGK	Fc-III-4C
